# Decoding the dynamics of cellular metabolism and the action of 3-bromopyruvate and 2-deoxyglucose using pulsed stable isotope-resolved metabolomics

**DOI:** 10.1186/2049-3002-2-9

**Published:** 2014-06-30

**Authors:** Matthias Pietzke, Christin Zasada, Susann Mudrich, Stefan Kempa

**Affiliations:** 1 Integrative Metabolomics and Proteomics, Berlin Institute of Medical Systems Biology/Max-Delbrueck Center for Molecular Medicine, Robert Rossle Street 10, Berlin 13125, Germany; 2 Present address: Faculté des Sciences, de la Technologie et de la Communication, University of Luxembourg, 162 A, Avenue de la Faïencerie L-1511, Luxembourg, Luxembourg

**Keywords:** Stable isotope labeling, GC-MS, Cancer metabolism, Glycolysis, Metabolomics, 3-Bromopyruvate, 2-Deoxyglucose

## Abstract

**Background:**

Cellular metabolism is highly dynamic and continuously adjusts to the physiological program of the cell. The regulation of metabolism appears at all biological levels: (post-) transcriptional, (post-) translational, and allosteric. This regulatory information is expressed in the metabolome, but in a complex manner. To decode such complex information, new methods are needed in order to facilitate dynamic metabolic characterization at high resolution.

**Results:**

Here, we describe pulsed stable isotope-resolved metabolomics (pSIRM) as a tool for the dynamic metabolic characterization of cellular metabolism. We have adapted gas chromatography-coupled mass spectrometric methods for metabolomic profiling and stable isotope-resolved metabolomics. In addition, we have improved robustness and reproducibility and implemented a strategy for the absolute quantification of metabolites.

**Conclusions:**

By way of examples, we have applied this methodology to characterize central carbon metabolism of a panel of cancer cell lines and to determine the mode of metabolic inhibition of glycolytic inhibitors in times ranging from minutes to hours. Using pSIRM, we observed that 2-deoxyglucose is a metabolic inhibitor, but does not directly act on the glycolytic cascade.

## Background

Central metabolism is highly flexible and continuously adjusts to the physiological program of the cell, organ, and organism. In a healthy state, cellular metabolism is tightly regulated to guarantee physiological function and the efficient usage of available resources. However, the underlying mechanisms leading to metabolic dysfunction are often poorly understood. To gain deeper insights into the dynamics of metabolism, techniques enabling a quantitative and time-resolved analysis of the metabolome are needed to allow a detailed real-time view of carbon routing through central carbon metabolism (CCM): glycolysis, pentose phosphate pathway (PPP), tricarboxylic acid cycle (TCA) cycle, and amino acid metabolism.

Metabolic flux is an expression of the activity of a network of enzymatic reactions. This network is influenced by several factors: the expression levels of the catalytic enzymes and the relative concentrations of substrates and products. In addition, allosteric regulation and post-translational modifications, including phosphorylation, ubiquitylation and, most recently recognized, acetylation, control metabolic activity. Metabolic flux cannot be inferred from transcript or protein levels of constituent enzymes, but rather requires direct measurement. Several techniques for metabolic flux analysis (MFA) have been established previously. Flux balance analysis (FBA) and stoichiometric MFA rely on the quantification of substrate uptake and product secretion of the cellular system over time [1,2]. Integration of further parameters, such as cell growth and biomass production, provides an overall quantification of metabolic performance.

However, such methods particularly fail in the case of parallel or circular metabolic pathways [3]. In addition, unlike bacteria, mammalian cells contain different compartments and are not optimized to reach maximum biosynthesis and growth rate, making calculations and modeling more complex and error-prone [4].

To solve these difficulties and improve the quality of measurements, isotopes were introduced into the monitored systems. In the early days of biochemistry, radioactive isotopes were used to assess the operation of the most important metabolic pathways: glycolysis [5], the TCA cycle [6], and the Calvin-Benson cycle [7]. As early as 1977, radioisotopes were applied to trace carbon flow throughout rat metabolism *in vivo*[8]. Recently, stable isotopes have been applied to humans [9]. Nowadays, the use of stable isotopes (e.g., carbon-13 or nitrogen-15) in combination with mass spectrometry or nuclear magnetic resonance allows a detailed analysis of metabolism [10]. Typically, a mixture of stable isotope-labeled substrates is fed to cells, producing complex isotope distribution patterns of metabolic intermediates [11,12]. The analysis of these patterns with mathematical models that consider the network structure and carbon transitions increases knowledge about metabolic activity and carbon routing within the cellular system [13]. Such studies have already given new insights into the regulation of the central metabolism by well-characterized oncogenes [14]. However, with increased sensitivity of recently developed analytical systems, it is now possible to detect ^13^C enrichment in free metabolites, instead of the more abundant protein-bound amino acids, thereby enabling a switch from stationary to instationary metabolic flux analysis [15].

Herein, we report the development of a strategy that allows for the direct measurement of dynamic metabolic activity through central carbon pathways using stable isotopes and mass spectrometry. The workflow presented is designed to achieve a comprehensive measurement of CCM with broad coverage of intermediates. Stable isotope-labeled substrates are applied to cells, and time-resolved isotope enrichment and quantification of downstream metabolites can be performed within a single measurement—we termed this workflow pulsed stable isotope-resolved metabolomics (pSIRM).

The concept is similar to the dynamic metabolic flux analysis introduced for microorganisms by Nöh and Wiechert [16]. Due to the instationary isotope incorporation, metabolites that are tightly connected to the introduced substrates will display a higher isotope incorporation than the metabolites that are more distant [17].

Assessing the dynamics of CCM in minute timeframes enabled the comparison of carbon routing in several commonly used cancer cell lines and exploration of the mode of action of the glycolytic inhibitors 3-bromopyruvate (BrPyr) or 2-deoxyglucose (2DG). Surprisingly, we observed that 2DG does not directly block glycolysis but rather interferes with phosphate and thus ATP metabolism. In summary, we show that the pSIRM approach facilitates the identification of metabolic dysregulation directly, without the need to separately calculate metabolic fluxes [18].

## Methods

### Chemicals

Stable isotope-labeled substrates were purchased at the following company: u-^13^C-glucose and ^13^C_1_-glucose are from Campro Scientific (Berlin, Germany). Extraction chemicals methanol and chloroform were products of Merck (Whitehouse Station, NJ, USA). All other chemicals were bought in highest quality at Sigma-Aldrich (St. Louis, MO, USA) unless otherwise noted.

### Cell culture

Dr. Ulrike Ziebold (MDC Berlin-Buch, T98G, HeLa, HCT-116) and Dr. Markus Landthaler (MDC Berlin-Buch, HEK293) kindly provided the cell lines. The cell lines were cultivated in glucose-free Dulbecco's modified Eagle's medium (DMEM; Invitrogen, Renfrew, Scotland) supplemented with 2.5 g/l glucose, 10% fetal bovine serum (Invitrogen), and 1% penicillin/streptomycin (Invitrogen) and cultivated at 37°C in 5% CO_2_. The cells were passaged with appropriate split ratios every 3 days. Viable cell numbers were determined by trypan blue staining (0.04%, Invitrogen) and automated counting (BioRad, Hercules, CA, USA).

### pSIRM experiment

#### *Cell culture*/*cell seeding*

The number of cells for plating was determined to avoid contact inhibitory effects during the experiment for each cell line and nutrient condition separately. After seeding, the cells were cultured for 2 or 3 days. During that time, cell culture media was replaced 24 and 4 h prior to harvest.

#### *Cell labeling*/*harvest*

The adherent growth behavior of the used cell lines allowed the labeling with ^13^C substrates directly on the cell culture dish. Therefore, the cell culture medium was replaced with pre-warmed full label medium containing all carbon sources and supplements like standard cell culture for a defined time range. One carbon source was substituted with its carbon-13 variant according to the setup of the experiment. Hereafter, the cells were quickly flushed with label buffer (140 mM NaCl, 5 mM HEPES (Roth, Karlsruhe, Germany) with pH 7.4, major carbon sources according full label media) to remove extracellular metabolites. Immediately, the cells were quenched with 5 ml -20°C cold 50% methanol (containing cinnamic acid (2 μg/ml)). The cells were scratched from the culture dish in the solvent, transferred into a 15-ml falcon, and stored on ice or at -25°C until proceeding with metabolite extraction. In the pSIRM experiments with an application of ^13^C substrates for less than 5 min, the cells were incubated in label buffer directly.

#### Metabolite extraction

Methanol-chloroform-water extraction provides an effective extraction and subsequent separation of lipid and polar intermediates. One milliliter chloroform was added to 5 ml of methanolic cell extracts, shaken for 30 min at 4°C, and centrifuged at maximum speed for 15 min for phase separation. Both phases were collected separately and dried under vacuum. The extracts were stored at -25°C.

### GC-MS analysis

Derivatization was carried out as described with modifications [19]. The dried cell extracts were dissolved in 20 μl of methoxyamine hydrochloride solution (Sigma, 40 mg/ml in pyridine (Roth)) and incubated for 90 min at 30°C with constant shaking followed by the addition of 80 μl of *N*-methyl-*N*-[trimethylsilyl]trifluoroacetamide (MSTFA; Machery-Nagel, Dueren, Germany) and incubation at 37°C for 45 min. The extracts were centrifuged for 10 min at 10,000 × *g*, and aliquots of 30 μl were transferred into glass vials (Th. Geyer, Berlin, Germany) for gas chromatography-mass spectrometry (GC-MS) measurement.

#### Retention index standard

Nine alkanes (n-decane, n-dodecane, n-pentadecane, n-octadecane, n-nonadecane, n-docosane, n-octacosane, n-dotriacontane, and n-hexatriacontane) were dissolved in hexane, combined at a final concentration of 2 mg/ml and stored at 4°C. Retention index standard was added to the solvent (MSTFA) at a final concentration of 2% (*v*/*v*) during derivatization.

#### Quantification standard

The quantification mixture was composed of 63 compounds (stock concentration 1 mg/ml, 20% MeOH). A dilution series from 1:1, 1:2, 1:5, 1:10, 1:20, 1:50, 1:100, and 1:200 was prepared, portioned, dried under vacuum, and stored at -20°C. One set of quantification standard was treated in parallel with cell extracts during derivatization and measured in technical replicates within an experiment.

#### GC-MS measurement

Metabolite analysis was performed on a gas chromatography coupled to time of flight mass spectrometer (Pegasus III- TOF-MS-System, LECO Corp., St. Joseph, MI, USA), complemented with an auto-sampler (MultiPurpose Sampler 2 XL, Gerstel, Mülheim an der Ruhr, Germany). The samples and quantification standards were injected in split mode (split 1:5, injection volume 1 μl) in a temperature-controlled injector (CAS4, Gerstel) with a baffled glass liner (Gerstel). The following temperature program was applied during sample injection: initial temperature of 80°C for 30 s followed by a ramp with 12°C/min to 120°C and a second ramp with 7°C/min to 300°C and final hold for 2 min. Gas chromatographic separation was performed on an Agilent 6890 N (Agilent, Santa Clara, CA, USA), equipped with a VF-5 ms column of 30-m length, 250-μm inner diameter, and 0.25-μm film thickness (Varian, Palo Alto, CA, USA). Helium was used as carrier gas with a flow rate of 1.2 ml/min. Gas chromatography was performed with the following temperature gradient: 2-min heating at 70°C, first temperature gradient with 5°C/min up to 120°C and hold for 30 s; subsequently, a second temperature increase of 7°C/min up to 350°C with a hold time of 2 min. The spectra were recorded in a mass range of 60 to 600 U with 20 spectra/s at a detector voltage of 1750 V.

#### Data analysis

The vendor software ChromaTOF Version 4.42 (LECO) was used for metabolite evaluation with the following parameters: baseline offset of 1, peak width of 4 s, signal/noise of 20, and peak smoothing of 11 data points. Retention indices were calculated based on retention index standards. The Golm Metabolome Database (GMD) provided mass spectra and retention information for peak identification [20]. The quantification routine of ChromaTOF was used for external calibration based on the measured quantification standards. Exported .txt files included the following: name, quantification mass retention index, first dimension retention time, second dimension retention time, area, concentration, match, reverse, quantitative signal/noise, type, concentration units, and the peak true spectrum in absolute values. For further data analysis, the tool MetMax was developed in cooperation with the MPIMP in Potsdam-Golm (http://gmd.mpimp-golm.mpg.de/apps/metmax) [21]. MetMax provided the extraction of peak areas and quantities (retention analysis mode) and intensities of pre-defined mass ranges (isotope concentrator mode) from the exported .txt files. The in-house-developed pSIRM-wizard enabled the determination of ^13^C-label incorporation based on the exported data following the descriptions and equations stated in the paper. The R package is available on request.

### Experimental procedures

#### Reproducibility

T98G cells were seeded (6.5 × 10^5^ cells/10-cm cell culture dish) in 10 ml DMEM (2.5 g/l glucose, 4 mM glutamine, 10% FBS, and 1% pen/strep) and cultivated for 3 days. Media changes were performed 24 and 4 h prior the harvest to avoid nutrient deprivation.

Stable isotope labeling with ^13^C glucose was applied at three independent dishes for 3 min. Cell harvest and extraction was carried out as described above. The extracts of all dishes were pooled, portioned, and measured six times to evaluate technical reproducibility. The biological variance was determined by the measurement of five individually handled dishes of T98G cells measured in four technical replicates. In addition, two plates of T98G cells were harvested with ^12^C glucose for the acquisition of reference spectra.

#### Quantification addition

Seven dishes of T98G cells were harvested as described above. The cells were extracted a second time with 20% (*v*/*v*) MeOH containing an internal standard (cinnamic acid, 2 μg/ml), and pooled. Quantification mixtures and quantification mixtures with spiked in cell extracts as well as cell extracts were measured, and recovery was calculated from these data.

#### Verification of calculation strategy by the measurement of known ratios of ^13^C_1_-Glc and ^12^C-Glc

One milligram per milliliter stock solutions of glucose and ^13^C_1_-glucose (Campro Scientific) were prepared in 20% MeOH and mixed in known ratios of 0%, 2%, 5%, 10%, 25%, 50%, 75%, 90%, 95%, 98%, and 100% of ^13^C_1_-glucose; three independent batches were prepared. Twenty microliters were dried under vacuum, derivatized, and measured as described above. Measurement and data extraction were performed as described and uncorrected, targeted, and position-independent strategies applied.

#### Metabolic profiling of cell lines

T98G, HEK293, HeLa, and HCT-116 were grown for analysis of the metabolic profile under identical nutrient conditions (DMEM, 10% FBS, 2.5 g/l glucose, 4 mM glutamine). Seeding densities were evaluated in advance to avoid contact inhibition. Labeling with ^13^C-glucose and cell harvest were performed as described. Three dishes for each cell line were pooled, processed as described above, and measured in technical replicates.

#### Glycolytic inhibition

T98G cells were seeded and harvested as described for the reproducibility experiment. Inhibitors were added in the following concentrations separately for 12 min: 2 mM BrPyr and 2, 4, and 10 mM 2DG. On a separate dish, 2 mM mannitol was applied as osmotic control. Subsequently, media were replaced with 5ml pre-warmed labeling buffer containing 2.5 g/l u-^13^C-glucose, 2 mM glutamine, and inhibitors for 3 min. Cell harvest, extraction, and metabolite measurement were performed as described above.

## Results

### Coverage of intermediates of CCM

**Figure 1:**
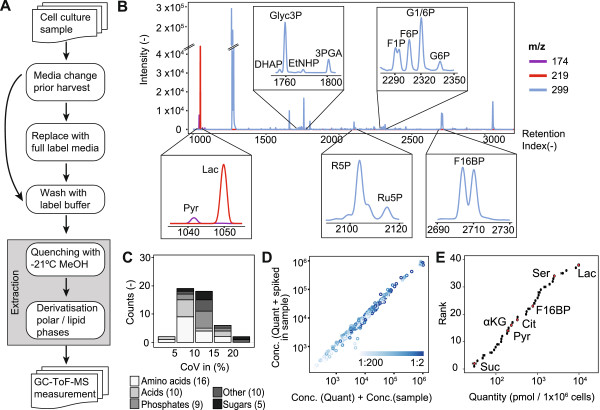
**pSIRM workflow including sample preparation, identification, and quantification of metabolites. (A)** Scheme of the experimental workflow from cell culture and cell harvest to GC-MS measurement. **(B)** A representative GC-MS selected ion chromatogram obtained from a cell culture sample using temperature-controlled and split injection. Due to the method, the peaks of pyruvate and lactate are baseline separated, detected, and quantified. The majority of phosphates are distinguishable by their retention behavior. **(C)** Distribution of the coefficient of variation (CoV) of the measured metabolite quantities from five biological replicates; distinct compound classes are indicated. **(D)** Spike-in experiment in order to test recovery of metabolites (see also Additional file 1: Table S5). Therefore, the samples were measured alone and with spiked-in quantification mixtures at seven dilutions. **(E)** Absolute quantities of metabolites of T98G cells ranked by concentrations.

CCM comprises a large number of small molecules differing in their chemical properties, making detection and quantification of all intermediates using a single technique challenging. We have further developed GC-MS-based metabolomics to measure the intermediates of CCM with broad coverage, in absolute quantities, and to extract isotope information from the fragment spectra. This improved workflow (Figure 1A) builds the basis for pSIRM. The GC-MS analysis follows the protocol introduced by Roessner-Tunali and co-workers [19] with adaptations indicated in Kempa et al. [21]. We further refined the chromatography in order to detect and quantify CCM intermediates. We specifically identified conditions for the reliable separation and quantification of pyruvate and lactate (Figure 1B). Further improvements were made using a thermo-regulated injector, resulting in the temperature-dependent transfer of compounds from the liner to the column, and by optimizing temperature program and gas flow (see ‘Methods’ section).

### Cell harvest strategy

The separation of mammalian cell cultures from nutrient-rich culture medium without disturbing the cellular metabolic homeostasis is crucial. Both cold and heat inactivation (quenching) of metabolism, with or without washing steps, have been reported previously [22-24].

To overcome these problems, we designed a cell-harvesting strategy aimed at preserving the metabolic homeostasis during harvest that involves (i) exchange of culture medium prior to pSIRM experiment (2–4 h) to minimize concentration changes of nutrients during stable isotope pulse labeling and (ii) the continuous supply of major nutrients during the whole harvest, preventing the disruption of glycolysis or glutaminolysis (Additional file 1: Figure S1).

Depending on the duration of incubation, ^13^C-substrates are either added to a full label medium containing all factors and nutrients or to a label buffer containing main nutrients (e.g., glucose and glutamine), buffers, and salts to maintain osmolality and pH. In both cases, one major nutrient is replaced by carbon-13 variant (Additional file 1: Table S1).

The use of label buffer for pSIRM experiments is appropriate for incubation times not longer than 5 min. For extended incubations, a full label medium is suggested. Adherent cell lines are labeled while growing attached to their plates. Growth medium is replaced with pre-warmed full label medium. After incubation, the cells are rinsed with label buffer for a few seconds. Immediate adding of pre-cold methanol (50% *v*/*v*, -20°C) quenches all cellular processes directly and initiates metabolite extraction (see ‘Methods’ section).

### Reproducibility

In order to test the reproducibility of metabolite quantification, T98G cells were harvested, pooled, and measured six times independently, thereby giving the technical variance of the method. Five independently grown plates were treated separately during harvest and labeling, and measured in three technical replicates to evaluate the biological reproducibility (Additional file 1: Figure S2). We observed that 80% of metabolites showed a technical variation below 15% (Figure 1C and Additional file 1: Table S2).

### Quantification of CCM metabolites

Absolute quantification of metabolites improves metabolomics analyses. It enables inter-batch and inter-laboratory comparison and is a prerequisite for quantitative modeling. Different methods for absolute quantification have been described: isotopic dilution is often applied for liquid chromatography-mass spectrometry (LC-MS)-based quantification [25]. This strategy enables correction for strong matrix effects as well as ion suppression. However, the experimentally introduced isotope-labeled substances may interfere with the measurement of isotopomer distributions for metabolic flux analyses, making it necessary to run different measurements for quantification and isotopomer extraction [26,27]. Electron impact ionization (EI) is the standard ionization technique of GC-MS. This harsh ionization allows monitoring even of uncharged molecules, such as alkanes, and suffers less from ion suppression. In order to quantify metabolites, we established calibration curves using known quantities of a mixture of metabolites, mimicking the composition of the cellular metabolome. This mixture is composed of 63 compounds (Additional file 1: Table S3) and is measured in eight different dilutions spanning 3 orders of magnitude, resulting in 73 calibration curves when considering multiple derivates. Out of these curves, 54 delivered a regression curve with *R*^2^ > 0.95. Recovery and quantification of metabolites are influenced by several factors [28]. To test the accuracy and concentration range suitable for quantification, we performed a ‘spike-in’ experiment. We thus measured the quantitative mixture and biological extracts separately as well as the biological extract with spiked-in quantitative mixture. The comparison of the derived concentration values demonstrates the reliability of our approach. The majority of the compounds showed similar curves which were shifted by the quantity found in the added biological samples (Figure 1D and Additional file 1: Figure S3, Table S4). The recovery of the quantified compounds is, on average, 111% (Additional file 1: Table S5)—from a typical cell culture, around 40 metabolites can be reliably quantified in absolute terms (Figure 1E and Additional file 1: Table S6).

### Determination of time-resolved isotope incorporation rates

**Figure 2:**
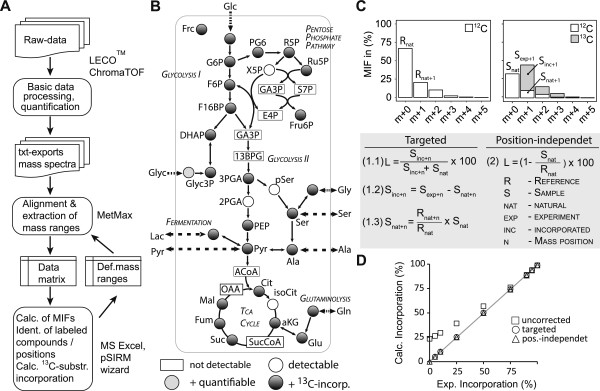
**pSIRM workflow including the determination and calculation of stable isotope incorporation. (A)** Data analysis workflow from GC-MS raw data up to quantification and the calculation of ^13^C-label incorporation. **(B)** Map of the central carbon metabolism highlighting detection and quantification of intermediates as well as evaluation of stable isotope enrichment after application of ^13^C-glucose or ^13^C-glutamine in cell culture experiments. **(C)** Visualization of mass isotopomer fractions (MIF) of a mass fragment of ^12^C-glucose (left) and a 1:1 mixture of ^12^C- and ^13^C_1_-glucose (right). Targeted (Formula 1.1–1.3) and position-independent (Formula 2) calculation strategies provide the determination of ^13^C-label incorporation (*L* in (%)). **(D)** Calculation strategies were validated by the analysis of known ratios of ^12^C-glucose: ^13^C_1_-glucose. Shown are expected versus measured stable isotope incorporation comparing both correction strategies.

The concept of mass isotopomer analysis [29,30] relies on the information about the incorporation of stable isotopes in the metabolite pools, which is inherited in the intensity shift within isotopomers. A prerequisite for a reliable analysis of isotopomers is the extraction of the corresponding mass spectra from GC-MS chromatograms. To determine the stable isotope incorporation in intermediates of CCM, we have established the pSIRM data analysis workflow (Figure 2A). With this approach, the quantity of a compound and the degree of stable isotope incorporation can be determined from the peak intensities and distribution of the mass isotopomers within the same measurement (Figure 2B). We compiled a list of mass pairs for the calculation of stable isotope incorporation based on our experimental results, partially overlapping with previously published data [28] (Additional file 1: Table S7).

To analyze the quantitative isotopomer distribution of the corresponding fragments, peak lists including the mass spectral information were generated with the vendor software ChromaTOF and exported as tab-separated .txt files. The MetMax Software [21] provides the extraction of intensities within pre-defined mass ranges from these txt files for the determination of MIF.

Mathematical approaches to calculate stable isotope enrichment [31] are already incorporated into existing software packages [32]. Usually, these approaches require the complete knowledge of the chemical composition of the molecular fragment analyzed. Here, we established a method to calculate stable isotope incorporation by subtracting natural occurring stable isotope abundances from experimentally derived mass spectra. This phenomenological approach does not require prior knowledge of the chemical composition of the fragments. Furthermore, this strategy takes into account concentration-dependent changes of the mass isotopomer distributions, which are laborious to calculate (Additional file 1: Figure S4). The incorporation of carbon-13 induces an intensity shift from the unlabeled position (*m* + 0) to the labeled position (*m* + *n*) corresponding to the number of incorporated carbon atoms. An example illustrating the incorporation of one carbon-13 is shown in Figure 2C. The ^13^C incorporation is defined as the ratio of the mass fraction at the position *m* + *n* of carbon-13 incorporation (*S*_exp + *n*_) to the sum of the intensities at *m* + 0 and *m* + *n* (*S*_nat_ + *S*_exp + *n*_). This simplified calculation leads to an overestimation of label incorporation due to naturally occurring carbon, nitrogen, and silicone isotopes in the compound and derivatization groups. The measurement of non-labeled reference spectra enables correcting for natural isotope abundance *S*_nat + *n*_ (Formula 1.3) and therefore to determine the actual ^13^C incorporation *S*_inc + *n*_ (Formula 1.2). Instead of the measurement-derived *S*_exp + *n*_, the use of the corrected *S*_inc + *n*_ provides a reliable estimate of ^13^C-carbon incorporation (Formula 1.1) in a position-dependent manner. The targeted strategy assumes a ^13^C incorporation at a single position, which holds true for most glycolytic intermediates after application of ^13^C_6_-glucose (Additional file 1: Table S7).

Incorporation of ^13^C-carbon atoms at multiple positions was addressed with an untargeted strategy. To calculate isotope incorporation into TCA cycle intermediates, we additionally employed a strategy to calculate the total label incorporation from the remaining intensity of the light fragment (*S*_nat_) compared to the overall intensity of the isotopomer (Formula 2). Therefore, the comparison of intensities of the ^12^C-fragments of the reference isotopomer (*R*_nat_) and sample isotopomer (*S*_nat_) enables the determination of label incorporation in a position-independent manner.

We verified the calculation strategies by mixing known amounts of ^13^C_1_-glucose with ^12^C-glucose and comparing the measured with the calculated isotope incorporation. The uncorrected strategy leads to an over-estimation of the label incorporation, as expected. The targeted and position-independent strategies reflected precisely the correct amount of ^13^C-label (Figure 2D and Additional file 1: Tables S8 and S9). Finally, we determined the label incorporation in T98G cells with technical and biological variation below 4% for the majority of metabolic intermediates (Additional file 1: Table S10).

### Application of pSIRM

**Figure 3:**
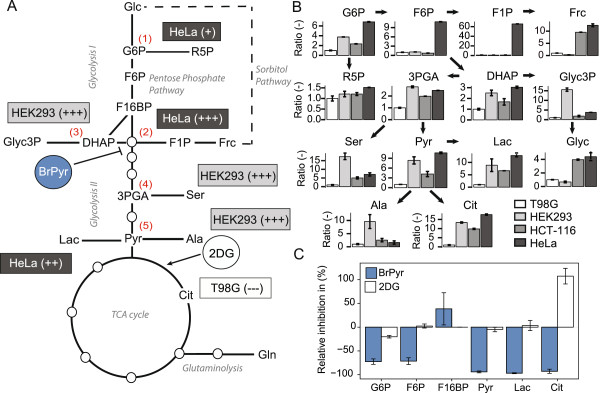
**Application of pSIRM for tracking carbon routing and monitoring influence of inhibitor treatment in cell cultures. (A)** Scheme of the central carbon metabolism (CCM) and branching points of glycolysis (1 to 5). Summarized are the results of the analyses of carbon routing in different cell lines and the impact of BrPyr or 2DG (T98G only) on CCM. **(B)** Quantification of ^13^C_6_-glucose incorporation within metabolic intermediates (labeled quantities) of T98G, HEK293, HeLa, and HCT-116 cells. Arrows between metabolites indicate links within the biochemical network. The data are presented relative to the T98G cells. **(C)** Visualization of the results of the pSIRM analysis of BrPyr- and 2DG-treated T98G cells.

Feeding cells with ^13^C-glucose allows monitoring of the activity of CCM, specifically of glycolysis and its branching points (Figure 3A) at the level of (1) G6P into the pentose phosphate pathway (PPP) and glycogen synthesis, (2) DHAP/GAP into PPP and (3) into Glyc3P (lipid synthesis), (4) 3PGA into serine and glycine metabolism, and (5) pyruvate into lactate, alanine or acetyl-CoA. The absolute concentration of ^13^C isotope-labeled intermediates was calculated for T98G cells (Additional file 1: Figure S5). Further, we compared four different cell lines (HeLa, HEK293, T98G, and HCT-116) cultivated under identical nutrient conditions fed with u-^13^C-glucose for 3 min. The labeled fractions of metabolites (the product of peak intensity multiplied with percentage of label incorporation) were compared relative to T98G cells (Figure 3B and Additional file 1: Table S11). Using this approach, clear differences among the tested cell lines were observed. HeLa cells showed the highest glycolytic activity. ^13^C isotope incorporation of fructose was detected in the HeLa and HEK293 cells, while ^13^C-labeled fructose-1-phosphate was detected only in the HeLa cells. The time-dependent label incorporation in ribose-5-phosphate, an important intermediate of the pentose phosphate pathway, was comparable among all cell lines—nearly identical for G6P, DHAP, Pyr, Lac, and Cit; only the T98G cells displayed a different labeling pattern. The HEK293 cells showed higher activity in amino acid synthesis as the labeled fractions of alanine and serine were increased. These results clearly demonstrate the ability of the presented method in order to highlight variations in carbon routing within different cellular systems.

We also analyzed the mode of action of two glycolytic inhibitors, 2DG and BrPyr. The T98G cells were treated for 12 min with each inhibitor (2 mM), followed by 3 min of ^13^C-glucose incorporation in the presence of each inhibitor (Figure 3C). The BrPyr treatment reduced the labeled fraction of G6P and F6P, whereas the total concentration of these compounds increased (Additional file 1: Table S7 and Table S12). The concentration of 3PGA diminished below the detection limit. Furthermore, downstream glycolysis was nearly completely inhibited (Figure 3C). The carbon flow into pyruvate, lactate, and citrate was reduced by around 100%, verifying specific suppression of GAPDH activity. The alterations within the glycolysis upstream may be explained by feedback inhibition of hexokinase by the accumulation of hexose phosphates. Further, lower concentrations of BrPyr reduced the carbon flow though glycolysis (Additional file 1: Figure S8). In comparison, 2 mM 2DG reduced the amount of the ^13^C fraction of hexose phosphates by 20%–30% and affected only marginally the isotope incorporation into pyruvate and lactate (Additional file 1: Table S13). Interestingly, ^13^C-glucose incorporation into citrate increased after 2DG treatment (Figure 3C). Increasing the 2DG concentration to 10 mM, a stronger inhibition was observed—mainly due to an overall decline of metabolite pool sizes rather than a decrease of ^13^C-label incorporation (Additional file 1: Figure S7).

## Discussion

Isotope pulse labeling is the ultimate tool to unravel metabolic pathways in plant and animal cells. The further development of analytical techniques such as mass spectrometry and nuclear magnetic resonance allows quantitative and structurally resolved analysis of stable isotope incorporation, thereby enabling a time-resolved analysis of known metabolic pathways and, potentially, the discovery of new connections within the metabolic network [33-35].

Here, we report that the pSIRM workflow integrates information about stable isotope incorporation and metabolomics analyses by introducing additional time-dependent information of the underlying pathway activities from a single measurement in a quantitative manner.

We have improved the cell-handling protocol, allowing for removal of abundant compounds from cultivation medium, and optimized sample preparation and GC-MS measurements. The combination of these improvements has created a reproducible analytical platform with a high coverage of CCM intermediates. Using GC-TOF-MS technology offers further advantages: the quantification of compounds suffers less from matrix effects due to the strong EI, thus enabling the application of external calibration for absolute quantification of 38 metabolites simultaneously in the T98G cells. Using external calibration instead of isotope dilution delivers advantages in all situations where the generation of sample material is limited, e.g., with primary cell lines or *in vivo* applications. The propagation of errors is minimized when quantities and isotope incorporation are measured from the same sample. Further, the untargeted character of TOF-MS allowed for the measurement of carbon-13 incorporation in an unbiased manner [36].

The pSIRM approach follows the concept of Nöh and Wiechert who proposed a distinct advantage of dynamic labeling in comparison to steady-state label incorporation [37]. Uniformly labeled ^13^C-substrates that do not yield information in stationary labeling can be used for dynamic labeling (Additional file 1: Figure S6), making analysis both less expensive and more generally applicable. Using pSIRM, we aimed to compare different metabolic states rather than perfect numerical description of the underlying fluxes. The functionality of this concept was demonstrated by decoding the metabolic basis of synthetic lethality induced by metabolic inhibition of senescent lymphoma cells [38] and in MYC-driven cancer cells after ARK5 inhibition [18].

Finally, dynamic labeling allowed the measurement of short-term changes within metabolism that could not be monitored by long-term label incorporation experiments. The application of pSIRM in combination with metabolic inhibitors or toxins may resolve the position of a drug target within the metabolic network. This is illustrated by the inhibition of glycolysis by BrPyr and by the failure of 2DG to acutely block glycolysis. In agreement with previous studies [39,40], we observed an increased concentration of hexose phosphates after BrPyr treatment (Additional file 1: Table S12), indicating that hexokinase is not the major target of this inhibitor. A strong decline of carbon flow downstream of GAPDH identifies this enzyme as target of BrPyr in agreement with literature data [41,42].

In comparison, 2DG did not block glycolysis directly under the conditions used. A 2 mM 2DG reduced the ^13^C-labeled fraction of hexose phosphates by only 20%–30% and did not affect the pyruvate or lactate levels. At the same time, a concentration- and time-dependent increase of 2-deoxyglucose-phosphate (2DG-P), resulting from phosphorylation by hexokinase, was detectable (Additional file 1: Figure S7).

2DG-P was found to accumulate inside the cells to high levels and exceeding the G6P concentrations by 10- to 20-fold (Additional file 1: Figure S4). As a response, cells adapted to 2DG treatment by increasing the carbon flow into citrate (Figure 3C), as reported previously [43,44]. From these results, we conclude that an accumulation of 2DG-P depletes the cellular phosphate pool by scavenging phosphate, thereby impairing ADP re-phosphorylation. Thus, ATP-dependent processes in the cell, such as glycolysis, may be slowed down. Indeed, Hoffmann and co-workers measured concentrations of ATP and G6P in HepG2 cells and found a ratio of 1:20 (G6P/ATP) [24]. Thus, the levels of 2DG-P in our experiments may be at the same levels as of ATP. Very high concentrations of 2DG (10 mM) reduced isotope incorporation in pyruvate up to 80%, while isotope incorporation rates in G6P were unaltered. Moreover, it has been shown that 2DG treatment interferes with glycosylation of proteins, inducing stress in the endoplasmic reticulum [45,46].

In summary, the analysis of metabolic reprogramming after application of metabolic inhibitors in acute time scales has the potential to improve metabolic research. The dissection of primary metabolic effects from secondary consequences is now possible. This will enable the evaluation of the direct impact of small molecules on metabolic pathways from metabolic changes provoked by transcriptional reprogramming. Thus, pSIRM has the potential to support quantitative and time-resolved understanding of metabolic regulation.

## Conclusions

In the present work, we describe a GC-MS-based strategy to analyze and quantify stable isotope incorporation into intermediates of central carbon metabolism. We have used pSIRM to analyze the acute effects induced by the addition of 2DG and BrPyr. By analyzing the action of these compounds on the glycolytic pathway of a cancer cell line, we observed that 2DG acts indirectly, perhaps due to the depletion of the intracellular phosphate pool. Methods designed for the quantification of stable isotope incorporation at an isotopic instationary state, such as pSIRM, will become very useful when analyzing the isotope incorporation *in vivo*. Ensuring an isotopic steady state for *in vivo* metabolic analysis is very time consuming and expensive. Although LC-MS-based methods for metabolomics analyses and quantification of isotope incorporation are widely used, GC-MS-based methods offer some advantages. The electron impact ionization is very harsh and allows a highly sensitive detection of less-charged molecules and a reliable quantification. Due to the sample preparation and the superior separation capacity of gas chromatography, compounds spanning a wide range of polarity can be separated. Specifically, the separation of stereoisomers of carbohydrates or organic acids, which are hardly distinguishable by LC-MS-based methods, can be achieved by GC-MS. The pSIRM workflow also has the advantage of allowing the assessment of ^13^C enrichment and metabolite abundance in the same sample. This simplifies the workflow for cell culture experiments and is prerequisite when analyzing metabolic dynamics *in vivo*.

## Abbreviations

3PGA: Glyceric acid-3-phosphate; Cit: Citrate; DHAP: Dihydroxyacetone phosphate; F6P: Fructose-6-phosphate; G6P: Glucose-6-phosphate by-product; Glyc3P: Glycerol-3-phosphate; Lac: Lactate; MeOH: Methanol.

## Competing interests

SK, CZ, and MP apply for a patent concerning the pSIRM workflow. There are no further competing or financial interests.

## Authors’ contributions

SK conceived the project. SK, MP, CZ, and SM designed the experiments. MP, CZ, and SM performed the experiments. MP, CZ, SM, and SK analyzed the data, and MP, CZ, and SK wrote the manuscript. All authors read and approved the final manuscript.

## Supplementary Material

Additional file 1 **Supplementary figures and tables.Figure S1.** Experimental setup of the harvesting procedure for a pSIRM cell culture experiment. **Figure S2.** Experimental scheme of the reproducibility experiment to determine technical and biological variances of GC-MS-derived data. **Figure S3.** Illustration of the quant addition experiment. **Figure S4.** Concentration dependency of mass isotopomer fractions. **Figure S5.** Absolute ^13^C quantities of intracellular metabolites in T98G cells. **Figure S6.** Time dependency of ^13^C label incorporation into intermediates of the central carbon metabolism. **Figure S7.** Graphical presentation of small-molecule inhibitor-induced rearrangement of central carbon metabolism. **Figure S8.** Concentration dependency of BrPyr-induced inhibition in carbon flow from ^13^C-glucose to lactate. **Figure S9.** Time- and concentration-dependent accumulation of 2-deoxyglucose-6-phosphate (2DG-P). **Table S1.** Composition of the full label medium and label buffer for pSIRM experiments used for the labeling of T98G cells and other cell lines as mentioned in the manuscript. **Table S2.** Biological and technical variation of the measurement of metabolite pool sizes in T98G cells. **Table S3.** Quant mixture composition and concentration range for each metabolite. **Table S4.** Data quant addition experiment. **Table S5.** Recovery of metabolites. **Table S6.** Intracellular metabolite concentrations of T98G cells. **Table S7.** Metabolite-specific mass fragments for the calculation of ^13^C isotope incorporation. **Table S8.** Illustration of the position-dependent strategy for correction of natural ^13^C isotope abundance. **Table S9.** Validation of correction strategies for the natural ^13^C-carbon abundance. **Table S10.** Biological and technical variation of ^13^C-glucose incorporation. **Table S11.** Comparison of carbon routing within the CCM of different cell lines. **Table S12.** 3-Bromopyruvate treatment rearranges central carbon metabolism in T98G cells. **Table S13.** Effect of 2-deoxyglucose on the central carbon metabolism of T98G cells. Click here for file

## References

[B1] Holms H Flux analysis and control of the central metabolic pathways in *Escherichia coli* FEMS Microbiol Rev 1996 19 2 85 116 8988566 10.1111/j.1574-6976.1996.tb00255.x

[B2] Holms H Flux analysis: a basic tool of microbial physiology Adv Microb Physiol 2001 45 271 340 11450111 10.1016/s0065-2911(01)45006-5

[B3] Wiechert W ^13^C metabolic flux analysis Metab Eng 2001 3 3 195 206 11461141 10.1006/mben.2001.0187

[B4] Zamboni N ^13^C metabolic flux analysis in complex systems Curr Opin Biotechnol 2011 22 1 103 108 20833526 10.1016/j.copbio.2010.08.009

[B5] Entner N Doudoroff M Glucose and gluconic acid oxidation of *Pseudomonas saccharophila* J Biol Chem 1952 196 2 853 862 12981024

[B6] Krebs HA Johnson WA The role of citric acid in intermediate metabolism in animal tissues Enzymologia 1937 4 148 156 10.4159/harvard.9780674366701.c143 6998725

[B7] Calvin M Benson AA The path of carbon in photosynthesis IV: the identity and sequence of the intermediates in sucrose synthesis Science 1949 109 2824 140 142 17759168 10.1126/science.109.2824.140

[B8] Kusaka M Ui M Tracer kinetic analysis of Cori cycle activity in the rat: effect of feeding Am J Physiol 1977 232 2 E136 E144 842621 10.1152/ajpendo.1977.232.2.E136

[B9] Fan TW Lane AN Higashi RM Farag MA Gao H Bousamra M Miller DM Altered regulation of metabolic pathways in human lung cancer discerned by ^13^C stable isotope-resolved metabolomics (SIRM) Mol Cancer 2009 8 41 19558692 10.1186/1476-4598-8-41 PMC2717907

[B10] Lane AN Fan TW Higashi RM Isotopomer-based metabolomic analysis by NMR and mass spectrometry Methods Cell Biol 2008 84 541 588 17964943 10.1016/S0091-679X(07)84018-0

[B11] Nanchen A Fuhrer T Sauer U Determination of metabolic flux ratios from ^13^C-experiments and gas chromatography-mass spectrometry data: protocol and principles Methods Mol Biol 2007 358 177 197 17035687 10.1007/978-1-59745-244-1_11

[B12] Szyperski T Glaser RW Hochuli M Fiaux J Sauer U Bailey JE Wuthrich K Bioreaction network topology and metabolic flux ratio analysis by biosynthetic fractional ^13^C labeling and two-dimensional NMR spectroscopy Metab Eng 1999 1 3 189 197 10937933 10.1006/mben.1999.0116

[B13] Sauer U Lasko DR Fiaux J Hochuli M Glaser R Szyperski T Wuthrich K Bailey JE Metabolic flux ratio analysis of genetic and environmental modulations of *Escherichia coli* central carbon metabolism J Bacteriol 1999 181 21 6679 6688 10542169 10.1128/jb.181.21.6679-6688.1999 PMC94132

[B14] Gaglio D Metallo CM Gameiro PA Hiller K Danna LS Balestrieri C Alberghina L Stephanopoulos G Chiaradonna F Oncogenic K-Ras decouples glucose and glutamine metabolism to support cancer cell growth Mol Syst Biol 2011 7 523 21847114 10.1038/msb.2011.56 PMC3202795

[B15] Wiechert W Noh K From stationary to instationary metabolic flux analysis Adv Biochem Eng Biotechnol 2005 92 145 172 15791936 10.1007/b98921

[B16] Nöh K Wiechert W Experimental design principles for isotopically instationary ^13^C labeling experiments Biotechnol Bioeng 2006 94 2 234 251 16598793 10.1002/bit.20803

[B17] Yuan J Fowler WU Kimball E Lu W Rabinowitz JD Kinetic flux profiling of nitrogen assimilation in *Escherichia coli* Nat Chem Biol 2006 2 10 529 530 16936719 10.1038/nchembio816

[B18] Liu L Ulbrich J Muller J Wustefeld T Aeberhard L Kress TR Muthalagu N Rycak L Rudalska R Moll R Kempa S Zender L Eilers M Murphy DJ Deregulated MYC expression induces dependence upon AMPK-related kinase 5 Nature 2012 483 7391 608 612 22460906 10.1038/nature10927

[B19] Roessner-Tunali U Hegemann B Lytovchenko A Carrari F Bruedigam C Granot D Fernie AR Metabolic profiling of transgenic tomato plants overexpressing hexokinase reveals that the influence of hexose phosphorylation diminishes during fruit development Plant Physiol 2003 133 1 84 99 12970477 10.1104/pp.103.023572 PMC196583

[B20] Kopka J Schauer N Krueger S Birkemeyer C Usadel B Bergmuller E Dormann P Weckwerth W Gibon Y Stitt M Willmitzer L Fernie AR Steinhauser D GMD@CSB.DB: the Golm Metabolome Database Bioinformatics 2005 21 8 1635 1638 15613389 10.1093/bioinformatics/bti236

[B21] Kempa S Hummel J Schwemmer T Pietzke M Strehmel N Wienkoop S Kopka J Weckwerth W An automated GCxGC-TOF-MS protocol for batch-wise extraction and alignment of mass isotopomer matrixes from differential ^13^C-labelling experiments: a case study for photoautotrophic-mixotrophic grown *Chlamydomonas reinhardtii* cells J Basic Microbiol 2009 49 1 82 91 19206143 10.1002/jobm.200800337

[B22] Munger J Bajad SU Coller HA Shenk T Rabinowitz JD Dynamics of the cellular metabolome during human cytomegalovirus infection PLoS Pathog 2006 2 12 e132 17173481 10.1371/journal.ppat.0020132 PMC1698944

[B23] Wahrheit J Niklas J Heinzle E Evaluation of sampling and quenching procedures for the analysis of intracellular metabolites in CHO suspension cells BMC Proc 2011 5 Suppl 8 82 10.1186/1753-6561-5-S8-P82 PMC3284895 22373310

[B24] Hofmann U Maier K Niebel A Vacun G Reuss M Mauch K Identification of metabolic fluxes in hepatic cells from transient ^13^C-labeling experiments: part I. Experimental observations Biotechnol Bioeng 2008 100 2 344 354 18095337 10.1002/bit.21747

[B25] Mashego MR Wu L Van Dam JC Ras C Vinke JL Van Winden WA Van Gulik WM Heijnen JJ MIRACLE: mass isotopomer ratio analysis of U-^13^C-labeled extracts. A new method for accurate quantification of changes in concentrations of intracellular metabolites Biotechnol Bioeng 2004 85 6 620 628 14966803 10.1002/bit.10907

[B26] Munger J Bennett BD Parikh A Feng XJ McArdle J Rabitz HA Shenk T Rabinowitz JD Systems-level metabolic flux profiling identifies fatty acid synthesis as a target for antiviral therapy Nat Biotechnol 2008 26 10 1179 1186 18820684 10.1038/nbt.1500 PMC2825756

[B27] Bennett BD Yuan J Kimball EH Rabinowitz JD Absolute quantitation of intracellular metabolite concentrations by an isotope ratio-based approach Nat Protoc 2008 3 8 1299 1311 18714298 10.1038/nprot.2008.107 PMC2710577

[B28] Vielhauer O Zakhartsev M Horn T Takors R Reuss M Simplified absolute metabolite quantification by gas chromatography-isotope dilution mass spectrometry on the basis of commercially available source material J Chromatogr B Analyt Technol Biomed Life Sci 2011 879 32 3859 3870 10.1016/j.jchromb.2011.10.036 22100557

[B29] Lin YY Cheng WB Wright CE Glucose metabolism in mammalian cells as determined by mass isotopomer analysis Anal Biochem 1993 209 2 267 273 8470798 10.1006/abio.1993.1118

[B30] Christensen B Nielsen J Isotopomer analysis using GC-MS Metab Eng 1999 1 4 282 290 10937821 10.1006/mben.1999.0117

[B31] van Winden WA Wittmann C Heinzle E Heijnen JJ Correcting mass isotopomer distributions for naturally occurring isotopes Biotechnol Bioeng 2002 80 4 477 479 12325156 10.1002/bit.10393

[B32] Zamboni N Fischer E Sauer U FiatFlux—a software for metabolic flux analysis from ^13^C-glucose experiments BMC Bioinform 2005 6 209 10.1186/1471-2105-6-209 PMC1199586 16122385

[B33] Metallo CM Gameiro PA Bell EL Mattaini KR Yang J Hiller K Jewell CM Johnson ZR Irvine DJ Guarente L Kelleher JK Vander Heiden MG Iliopoulos O Stephanopoulos G Reductive glutamine metabolism by IDH1 mediates lipogenesis under hypoxia Nature 2011 481 7381 380 384 22101433 10.1038/nature10602 PMC3710581

[B34] Hiller K Metallo C Stephanopoulos G Elucidation of cellular metabolism via metabolomics and stable-isotope assisted metabolomics Curr Pharm Biotechnol 2011 12 7 1075 1086 21466455 10.2174/138920111795909096

[B35] Keibler MA Fendt SM Stephanopoulos G Expanding the concepts and tools of metabolic engineering to elucidate cancer metabolism Biotechnol Prog 2012 28 6 1409 1418 22961737 10.1002/btpr.1629 PMC3586222

[B36] Hiller K Metallo CM Kelleher JK Stephanopoulos G Nontargeted elucidation of metabolic pathways using stable-isotope tracers and mass spectrometry Anal Chem 2010 82 15 6621 6628 20608743 10.1021/ac1011574

[B37] Nöh K Wiechert W The benefits of being transient: isotope-based metabolic flux analysis at the short time scale Appl Microbiol Biotechnol 2011 91 5 1247 1265 21732247 10.1007/s00253-011-3390-4

[B38] Dorr JR Yu Y Milanovic M Beuster G Zasada C Dabritz JH Lisec J Lenze D Gerhardt A Schleicher K Kratzat S Purfürst B Walenta S Mueller-Klieser W Gräler M Hummel M Keller U Buck AK Dörken B Willmitzer L Reimann M Kempa S Lee S Schmitt CA Synthetic lethal metabolic targeting of cellular senescence in cancer therapy Nature 2013 501 7467 421 425 23945590 10.1038/nature12437

[B39] da Silva AP P El-Bacha T Kyaw N dos Santos RS da-Silva WS Almeida FC Da Poian AT Galina A Inhibition of energy-producing pathways of HepG2 cells by 3-bromopyruvate Biochem J 2009 417 3 717 726 18945211 10.1042/BJ20080805

[B40] Birsoy K Wang T Possemato R Yilmaz OH Koch CE Chen WW Hutchins AW Gultekin Y Peterson TR Carette JE Brummelkamp TR Clish CB Sabatini DM MCT1-mediated transport of a toxic molecule is an effective strategy for targeting glycolytic tumors Nat Genet 2013 45 1 104 108 23202129 10.1038/ng.2471 PMC3530647

[B41] Ganapathy-Kanniappan S Geschwind JF Kunjithapatham R Buijs M Vossen JA Tchernyshyov I Cole RN Syed LH Rao PP Ota S Vali M Glyceraldehyde-3-phosphate dehydrogenase (GAPDH) is pyruvylated during 3-bromopyruvate mediated cancer cell death Anticancer Res 2009 29 12 4909 4918 20044597 PMC3743725

[B42] Barnard JP Reynafarje B Pedersen PL Glucose catabolism in African trypanosomes. Evidence that the terminal step is catalyzed by a pyruvate transporter capable of facilitating uptake of toxic analogs J Biol Chem 1993 268 5 3654 3661 8429041

[B43] Sauermann G Der Einfluss von 2-deoxy-D-glucose auf die glucoseoxydation in ascites-tumor-zellen Z Krebsforsch 1967 69 1 44 50 4171689 10.1007/BF00571740

[B44] Letnansky K Seelich F Über die beeinflussung von reaktionen des citronensäure-cyclus durch 2-deoxy-D-glucose Z Krebsforsch 1960 64 1 6 13761232

[B45] Kurtoglu M Gao N Shang J Maher JC Lehrman MA Wangpaichitr M Savaraj N Lane AN Lampidis TJ Under normoxia, 2-deoxy-D-glucose elicits cell death in select tumor types not by inhibition of glycolysis but by interfering with N-linked glycosylation Mol Cancer Ther 2007 6 11 3049 3058 18025288 10.1158/1535-7163.MCT-07-0310

[B46] Kang HT Hwang ES 2-Deoxyglucose: an anticancer and antiviral therapeutic, but not anymore a low glucose mimetic Life Sci 2006 78 12 1392 1399 16111712 10.1016/j.lfs.2005.07.001

