# Supplementary material for: Decoding the dynamics of cellular metabolism and the action of 3-bromopyruvate and 2-deoxyglucose using pulsed stable isotope-resolved metabolomics
Source: Cancer Metab. 2014 Jun 30;2:9. doi: 10.1186/2049-3002-2-9 (PMC4101711; doi:10.1186/2049-3002-2-9)
Supplement: Additional file 1 — Supplementary figures and tables.Figure S1. Experimental setup of the harvesting procedure for a pSIRM cell culture experiment. Figure S2. Experimental scheme of the reproducibility experiment to determine technical and biological variances of GC-MS-derived data. Figure S3. Illustration of the quant addition experiment. Figure S4. Concentration dependency of mass isotopomer fractions. Figure S5. Absolute 13C quantities of intracellular metabolites in T98G cells. Figure S6. Time dependency of 13C label incorporation into intermediates of the central carbon metabolism. Figure S7. Graphical presentation of small-molecule inhibitor-induced rearrangement of central carbon metabolism. Figure S8. Concentration dependency of BrPyr-induced inhibition in carbon flow from 13C-glucose to lactate. Figure S9. Time- and concentration-dependent accumulation of 2-deoxyglucose-6-phosphate (2DG-P). Table S1. Composition of the full label medium and label buffer for pSIRM experiments used for the labeling of T98G cells and other cell lines as mentioned in the manuscript. Table S2. Biological and technical variation of the measurement of metabolite pool sizes in T98G cells. Table S3. Quant mixture composition and concentration range for each metabolite. Table S4. Data quant addition experiment. Table S5. Recovery of metabolites. Table S6. Intracellular metabolite concentrations of T98G cells. Table S7. Metabolite-specific mass fragments for the calculation of 13C isotope incorporation. Table S8. Illustration of the position-dependent strategy for correction of natural 13C isotope abundance. Table S9. Validation of correction strategies for the natural 13C-carbon abundance. Table S10. Biological and technical variation of 13C-glucose incorporation. Table S11. Comparison of carbon routing within the CCM of different cell lines. Table S12. 3-Bromopyruvate treatment rearranges central carbon metabolism in T98G cells. Table S13. Effect of 2-deoxyglucose on the central carbon metabolism of T9 [file 2049-3002-2-9-S1.pdf]

**Additional file 1**
Decoding the dynamics of cellular metabolism and the action of 3-bromopyruvate and 2-deoxyglucose using pulsed stable isotope-resolved metabolomics
Pietzke and Zasada et al., Cancer and Metabolism 2014

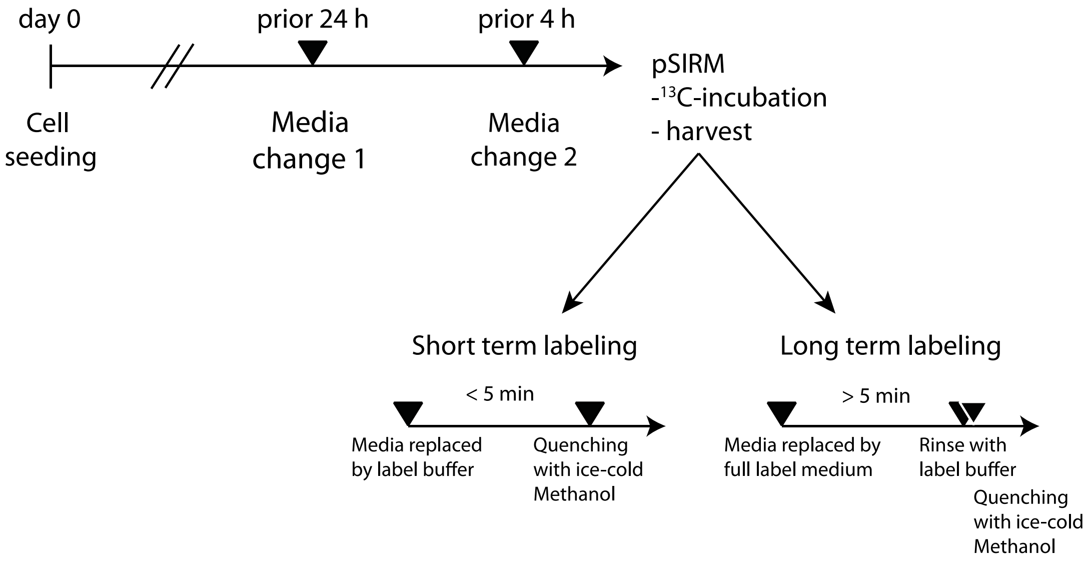


**Figure S 1 -** Experimental setup of harvesting procedure for a pSIRM cell culture experiment.

Cells are seeded with pre-determined cell number to avoid growth inhibitory effects until harvest on day 3 of the experiment. Media is replaced to supply cells with nutrients 24 and 4 hours prior the harvest. The harvest protocol summarizes the labeling procedure of adherent cell cultures (see Methods section).

**Additional file 1**
Decoding the dynamics of cellular metabolism and the action of 3-bromopyruvate and 2-deoxyglucose using pulsed stable isotope-resolved metabolomics
Pietzke and Zasada et al., Cancer and Metabolism 2014

**Table S 1** – Composition of the full label medium and label buffer for pSIRM experiments used for the labeling of T98G cells and other cell lines as mentioned in the manuscript.

| | Full label medium | Label buffer |
|---|---|---|
| **Base** | Dulbeccos modified eagle medium (DMEM)<br>- without Glucose<br>- without Glutamine<br>- without Pyruvate | HEPES (5 mM)<br>NaCl (140 mM)<br>pH 7.4 |
| **Carbon sources** | + $^{13}$C-Glucose (2.5 g/L)<br>+ $^{12}$C-Glutamine (4 mM) | + $^{13}$C-Glucose (2.5 g/L)<br>+ $^{12}$C-Glutamine (4 mM) |
| **Supplemented with** | + small molecules (inhibitor, antibiotics etc.) | |

**Additional file 1**
Decoding the dynamics of cellular metabolism and the action of 3-bromopyruvate and 2-deoxyglucose using pulsed stable isotope-resolved metabolomics
Pietzke and Zasada et al., Cancer and Metabolism 2014

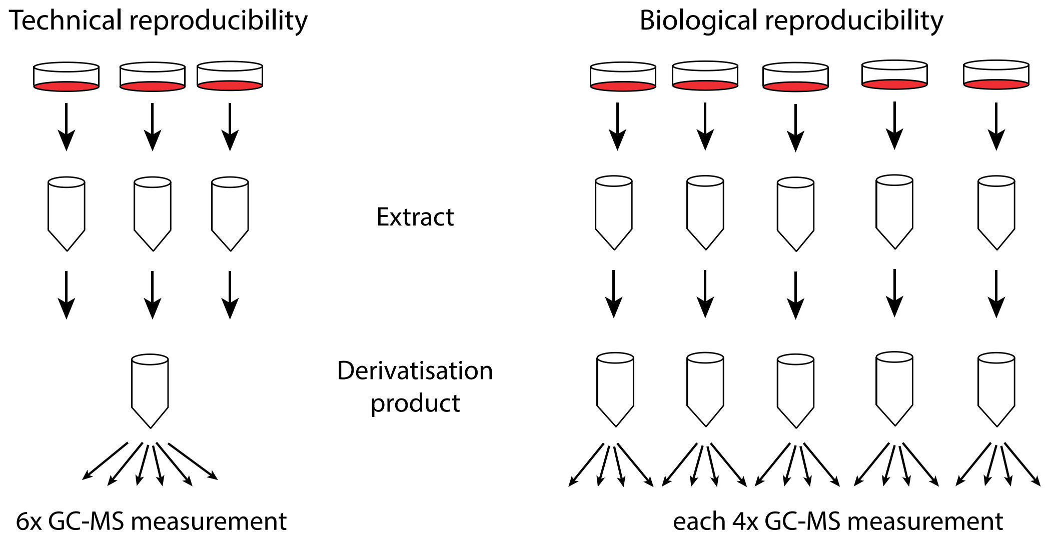


**Figure S 2 –** Experimental scheme of the reproducibility experiment to determine technical and biological variances of GC-MS derived data.
T98G cells were cultured for three days, and labeled with $^{13}C$-glucose for 3 minutes. Cell extracts were prepared separately for each cell culture plate. Technical variance was determined by the repeated measurement of pooled cell samples. Biological reproducibility was determined by repeated measurement of five independently treated cell samples.

**Additional file 1**
Decoding the dynamics of cellular metabolism and the action of 3-bromopyruvate and 2-deoxyglucose using pulsed stable isotope-resolved metabolomics
Pietzke and Zasada et al., Cancer and Metabolism 2014

**Table S 2 –** Biological and technical variation of the measurement of metabolite pool sizes in T98G cells.
The coefficient of variation (CoV) of the peak area for each metabolite was calculated from six consecutive measured technical replicates. The calculation of CoVs for the biological reproducibility based on five biological replicates, each measured in four technical repetitions.

| | **Coefficient of variation** | |
|---|---|---|
| **Compound** | **Technical replicates** | **Biological replicates** |
| Adenine | 13.7 | 14.7 |
| Alanine, beta- | 8.9 | 12.4 |
| Alanine | 6.8 | 10.0 |
| Asparagine | 9.0 | 5.3 |
| Aspartic acid | 3.6 | 10.3 |
| Butanoic acid, 2-amino- | 9.3 | 5.5 |
| Butanoic acid, 3-hydroxy- | 7.6 | 15.9 |
| Butanoic acid, 4-amino- | 16.1 | 19.6 |
| Citric acid | 8.9 | 10.6 |
| Cytosine | 15.1 | 12.1 |
| Fructose-1,6-bisphosphate | 15.3 | 15.6 |
| Fructose | 11.5 | 13.9 |
| Fructose-6-phosphate | 6.4 | 10.3 |
| Fumaric acid | 9.0 | 10.4 |
| Glucose | 8.6 | 7.0 |
| Glucose-6-phosphate | 10.8 | 11.6 |
| Glutamine | 5.9 | 11.9 |
| Glutaric acid, 2-hydroxy- | 8.7 | 13.4 |
| Glutaric acid, 2-oxo- | 12.2 | 14.3 |
| Glyceric acid | 6.3 | 7.1 |
| Glyceric acid-3-phosphate | 11.3 | 14.7 |
| Glycerol | 15.3 | 16.5 |
| Glycerol-3-phosphate | 10.2 | 13.1 |
| Glycine | 12.7 | 6.6 |
| Inositol, myo- | 11.3 | 11.6 |
| Isoleucine | 8.1 | 12.0 |
| Lactic acid | 3.9 | 11.0 |
| Leucine | 7.6 | 8.6 |
| Lysine | 12.0 | 11.5 |
| Malic acid | 9.2 | 13.3 |
| Methionine | 16.8 | 20.7 |
| Pantothenic acid | 11.2 | 11.2 |
| Phosphoenolpyruvic acid | 11.8 | 13.2 |
| Proline | 7.6 | 5.2 |
| Putrescine | 14.5 | 8.1 |
| Pyroglutamic acid | 10.1 | 13.8 |
| Pyruvic acid | 5.3 | 7.5 |
| Ribitol | 11.5 | 9.3 |
| Ribose-5-phosphate | 11.3 | 11.2 |
| Serine | 10.0 | 10.2 |
| Succinic acid | 15.0 | 21.3 |
| Sucrose | 23.2 | 29.5 |
| Threonine | 10.4 | 11.1 |
| Tyrosine | 10.7 | 8.2 |
| Uracil | 10.0 | 20.5 |
| Uridine 5'-monophosphate | 6.8 | 14.3 |
| Uridine | 21.5 | 13.5 |
| Valine | 5.6 | 11.6 |

**Table S 3** – Quant mixture composition and concentration range for each metabolite

| Compound | Concentration range in Quant mix (pmol) | |
|---|---|---|
| | Minimum | Maximum |
| Adenine | 37 | 7400 |
| Adenosine | 94 | 18709 |
| Alanine | 673 | 134695 |
| Alanine, beta | 56 | 11225 |
| Arginine | 57 | 11481 |
| Asparagine | 114 | 22707 |
| Aspartic acid | 75 | 15025 |
| Butyric acid, 3-hydroxy | 144 | 28818 |
| Butyric acid, 4-amino | 48 | 9697 |
| Citric acid | 260 | 52051 |
| Creatinine | 221 | 44201 |
| Cysteine | 41 | 8254 |
| Cytosine | 45 | 9001 |
| Dihydroxyacetone phosphate | 441 | 88183 |
| Erythritol, meso | 409 | 81887 |
| Fructose | 416 | 83259 |
| Fructose-1,6-bisphosphate | 271 | 54288 |
| Fructose-6-phosphate | 66 | 13154 |
| Fumaric acid | 172 | 34462 |
| Gluconic acid-6-phosphate | 73 | 14616 |
| Glucosamine | 23 | 4638 |
| Glucose | 1943 | 388544 |
| Glucose 1-phosphate | 59 | 11894 |
| Glucose-6-phosphate | 164 | 32884 |
| Glutamic acid | 680 | 135934 |
| Glutamine | 684 | 136855 |
| Glutaric acid | 151 | 30278 |
| Glutaric acid, 2-hydroxy | 286 | 57268 |
| Glutaric acid, 2-oxo | 171 | 34223 |
| Glyceraldehyde-3-phosphate | 323 | 64668 |
| Glyceric acid | 80 | 15987 |
| Glyceric acid-3-phosphate | 217 | 43478 |
| Glycerol | 326 | 65154 |
| Glycerol-3-phosphate | 135 | 26996 |
| Glycine | 333 | 66605 |
| GMP | 101 | 20113 |
| Hypotaurine | 92 | 18323 |
| Inosine | 56 | 11184 |
| Inositol, myo | 278 | 55506 |
| Isoleucine | 191 | 38116 |
| Lactic Acid | 2231 | 446190 |
| Leucine | 457 | 91484 |
| Lysine | 103 | 20531 |
| Malic acid | 224 | 44746 |
| Methionine | 34 | 6702 |
| Pantothenic acid | 84 | 16788 |
| Phenylalanine | 242 | 48429 |
| Phosphoenolpyruvic acid | 75 | 15036 |
| Proline | 304 | 60801 |
| Putrescine | 31 | 6208 |
| Pyroglutamic acid | 465 | 92937 |

| | | |
|---|---|---|
| Pyruvate | 1454 | 290803 |
| Ribose | 100 | 19983 |
| Ribose, 2-deoxy | 37 | 7455 |
| Ribose 5-phosphate | 456 | 91208 |
| Serine | 571 | 114188 |
| Succinic acid | 212 | 42341 |
| Threonine | 839 | 167898 |
| Tryptophan | 49 | 9793 |
| Tyrosine | 55 | 11038 |
| Uracil | 134 | 26764 |
| Uridine 5′-monophosphate | 407 | 81489 |
| Valine | 213 | 42680 |

**Additional file 1**
Decoding the dynamics of cellular metabolism and the action of 3-bromopyruvate and 2-deoxyglucose using pulsed stable isotope-resolved metabolomics
Pietzke and Zasada et al., Cancer and Metabolism 2014

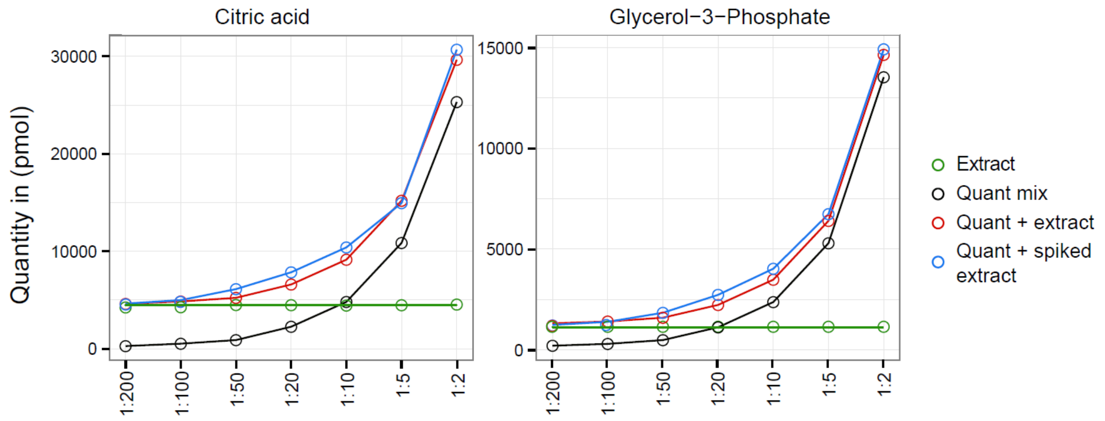


**Figure S 3 -** Illustration of quant-addition experiment**.**
The measurement of the quant mixture delivers calibration curves, which were used to quantify the biological sample – here shown for citric acid and glycerol-3-phosphate. The quantification results are shown for the individual measurements of quantification mixtures (black), cell samples (green), the sum of quantities of cell extracts and quantification mixtures (red), and the measurements of samples spiked into quantification mixtures (blue).

**Additional file 1**

Decoding the dynamics of cellular metabolism and the action of 3-bromopyruvate and 2-deoxyglucose using pulsed stable isotope-resolved metabolomics

Pietzke and Zasada et al., Cancer and Metabolism 2014

**Table S 4** – Data quant addition experiment. T98G cell extracts, equivalent to 7.14 x $10^5$ cells, were quantified by external quantification. Same amounts of extracts were spiked into each dilution of the quantification mixture.

| Compound | Concentration in extract in pmol (Avg ± SD) | Conc. quant (measured) + conc. extract (pmol) | | | | | | | Conc. (Quant + spiked in extract) (pmol) | | | | | | |
|---|---|---|---|---|---|---|---|---|---|---|---|---|---|---|---|
| | | 1:200 | 1:100 | 1:50 | 1:20 | 1:10 | 1:5 | 1:2 | 1:200 | 1:100 | 1:50 | 1:20 | 1:10 | 1:5 | 1:2 |
| Adenine | 614 ± 182 | 614 | 614 | 807 | 950 | 1371 | 2184 | 4220 | 697 | 576 | 791 | 1128 | 1466 | 2032 | 4422 |
| Alanine, beta- | 2300 ± 624 | 2300 | 2434 | 2560 | 2883 | 3456 | 4497 | 8130 | 2078 | 1987 | 2275 | 2722 | 3115 | 4105 | 6419 |
| Alanine | 9840 ± 3183 | 10689 | 11537 | 12291 | 16541 | 24554 | 36271 | 83215 | 11637 | 8756 | 11316 | 15642 | 22001 | 34702 | 83667 |
| Butanoic acid, 3-hydroxy- | 213 ± 88 | 370 | 529 | 780 | 1717 | 3263 | 5977 | 16149 | 458 | 507 | 1000 | 2238 | 4209 | 7850 | 19883 |
| Citric acid | 4333 ± 330 | 4609 | 4855 | 5225 | 6596 | 9141 | 15188 | 29647 | 4635 | 4992 | 6119 | 7847 | 10398 | 14948 | 30685 |
| Cytosine | 1467 ± 326 | 1467 | 1753 | 1857 | 1994 | 2347 | 3224 | 5995 | 1492 | 1585 | 1975 | 2649 | 3233 | 4266 | 7492 |
| Erythritol | 1403 ± 147 | 1869 | 2266 | 2987 | 5228 | 9093 | 17585 | 43782 | 1813 | 2390 | 3651 | 6710 | 11466 | 21274 | 49021 |
| Fructose BP | 9224 ± 473 | 9678 | 10060 | 10707 | 12880 | 17166 | 26803 | 49782 | 9809 | 10729 | 12542 | 15474 | 19566 | 27418 | 50961 |
| Fructose MP | 8791 ± 793 | 9225 | 9615 | 10287 | 12481 | 16838 | 26351 | 50400 | 9334 | 10040 | 11675 | 14497 | 18623 | 26486 | 51298 |
| Fructose-6-phosphate | 326 ± 37 | 457 | 533 | 613 | 912 | 1501 | 2980 | 7169 | 311 | 363 | 436 | 723 | 1118 | 2030 | 4996 |
| Fumaric acid | 1234 ± 93 | 1431 | 1617 | 1929 | 2848 | 4585 | 8242 | 18609 | 1462 | 1744 | 2357 | 3621 | 5852 | 9728 | 20213 |
| Glucose-1/6-phosphate | 1137 ± 207 | 1494 | 1613 | 1915 | 2881 | 4954 | 10100 | 24578 | 1332 | 1489 | 1837 | 3155 | 5141 | 8372 | 19299 |
| Glutaric acid, 2-hydroxy- | 396 ± 25 | 680 | 960 | 1531 | 3345 | 6495 | 12605 | 29013 | 783 | 1277 | 2410 | 5245 | 9134 | 15837 | 33714 |
| Glutaric acid, 2-oxo- | 6150 ± 380 | 6150 | 6529 | 6787 | 7852 | 9624 | 13348 | 22607 | 6284 | 6766 | 7550 | 8952 | 10693 | 13739 | 22961 |
| Glutaric acid | 136 ± 51 | 265 | 443 | 733 | 1618 | 3248 | 6360 | 15488 | 317 | 561 | 1002 | 2319 | 4442 | 8281 | 18164 |
| Glyceric acid | 223 ± 24 | 299 | 377 | 521 | 1001 | 1792 | 3463 | 8443 | 309 | 439 | 696 | 1398 | 2476 | 4456 | 9674 |
| Glyceric acid-3-phosphate | 875 ± 139 | 875 | 1436 | 1628 | 2795 | 4864 | 9482 | 22350 | 1090 | 1196 | 1697 | 2860 | 4455 | 7944 | 19644 |
| Glycerol | 4305 ± 369 | 4568 | 4981 | 5732 | 7979 | 11706 | 17908 | 37709 | 4785 | 5242 | 6415 | 9767 | 13930 | 22057 | 45069 |
| Glycerol-3-phosphate | 1111 ± 142 | 1315 | 1406 | 1594 | 2228 | 3482 | 6402 | 14642 | 1236 | 1389 | 1839 | 2729 | 4025 | 6736 | 14917 |
| Glycine | 12759 ± 3200 | 13085 | 13507 | 14327 | 16497 | 20261 | 26574 | 46330 | 12253 | 10957 | 12341 | 15094 | 17442 | 22535 | 36278 |
| Hypotaurine | 2551 ± 273 | 2665 | 2770 | 2913 | 3438 | 4502 | 6506 | 11552 | 2617 | 2751 | 3241 | 4034 | 5103 | 6938 | 12927 |
| Inositol, myo- | 7313 ± 885 | 7655 | 7878 | 8301 | 9637 | 12310 | 18742 | 35335 | 7811 | 8279 | 9033 | 11657 | 14604 | 21199 | 39071 |
| Lactic acid | 189454 ± 18268 | 192498 | 194432 | 197362 | 208360 | 229315 | 280861 | 432389 | 194868 | 195966 | 218580 | 246012 | 269882 | 310055 | 424055 |
| Leucine | 16789 ± 3287 | 16789 | 17776 | 18899 | 22799 | 28801 | 35738 | 63458 | 18069 | 16244 | 20882 | 25246 | 30916 | 37820 | 63263 |
| Malic acid | 5428 ± 321 | 5676 | 5884 | 6262 | 7462 | 9561 | 14172 | 27697 | 5682 | 6436 | 7568 | 9418 | 12320 | 17703 | 32113 |
| Pantothenic acid | 1396 ± 244 | 1488 | 1569 | 1715 | 2175 | 2990 | 4917 | 9710 | 1597 | 1740 | 2090 | 3099 | 4038 | 6077 | 11764 |
| Phenylalanine | 10229 ± 2156 | 10632 | 10823 | 11110 | 12534 | 15479 | 20126 | 35598 | 10559 | 10023 | 11674 | 13275 | 15093 | 17457 | 34055 |
| Proline | 7661 ± 1824 | 7661 | 7962 | 8766 | 12088 | 17110 | 20495 | 39253 | 8525 | 7189 | 10088 | 13832 | 18334 | 24202 | 43926 |
| Putrescine | 151 ± 12 | 198 | 238 | 311 | 499 | 823 | 1412 | 3415 | 186 | 221 | 325 | 552 | 872 | 1552 | 3353 |
| Pyroglutamic acid | 58365 ± 4119 | 58818 | 59351 | 60187 | 62939 | 67892 | 77985 | 105173 | 56276 | 61889 | 68508 | 76717 | 83975 | 97376 | 125674 |
| Pyruvic acid | 31775 ± 5243 | 32523 | 35292 | 38762 | 48600 | 66948 | 95473 | 178251 | 35120 | 35723 | 44233 | 64988 | 98394 | 141628 | 259046 |
| Ribose | 91 ± 27 | 183 | 289 | 502 | 1086 | 2114 | 4566 | 9154 | 230 | 347 | 656 | 1313 | 2422 | 4185 | 8674 |
| Serine | 23025 ± 3328 | 23674 | 24376 | 25174 | 28578 | 35000 | 45122 | 82883 | 24334 | 24116 | 28823 | 35298 | 42207 | 53976 | 93783 |
| Succinic acid | 1801 ± 134 | 2011 | 2260 | 2679 | 4098 | 6439 | 10545 | 23352 | 2040 | 2373 | 3233 | 5268 | 8186 | 13249 | 26145 |
| Threonine | 36780 ± 5628 | 37576 | 38779 | 40274 | 45846 | 56066 | 71285 | 125978 | 38110 | 37739 | 45000 | 55794 | 67967 | 91359 | 158356 |
| Uracil | 4135 ± 2397 | 4135 | 4403 | 4722 | 5428 | 6721 | 9606 | 17499 | 2044 | 1258 | 900 | 1887 | 4457 | 6908 | 14402 |
| Valine | 11337 ± 2111 | 11559 | 11847 | 12214 | 13616 | 15970 | 19668 | 35039 | 11936 | 11267 | 14342 | 16885 | 19559 | 23046 | 33794 |

**Table S 5** - Recovery of metabolites

The recovery is defined as ratio of the quantity of spike-in and summed up quantities of individual measured quantification mixtures and extracts. The recovery was calculated at equal concentrations of quantification mixture and extracts.

| **Compound** | **Recovery (%)** |
|---|---|
| Adenine | 112.9 |
| Alanine, beta- | 91.3 |
| Alanine | 92.1 |
| Butanoic acid, 3-hydroxy- | 109.8 |
| Cinnamic acid, trans- | 104.7 |
| Citric acid | 116.4 |
| Cytosine | 132.3 |
| Erythritol | 122.2 |
| Fructose BP | 108.1 |
| Fructose MP | 110.6 |
| Fructose-6-phosphate | 75.3 |
| Fumaric acid | 102.2 |
| Glucose-1/6-phosphate | 102.7 |
| Glutaric acid, 2-hydroxy- | 124.1 |
| Glutaric acid, 2-oxo- | 107.0 |
| Glutaric acid | 119.6 |
| Glyceric acid | 125.0 |
| Glyceric acid-3-phosphate | 104.2 |
| Glycerol | 111.9 |
| Glycerol-3-phosphate | 122.5 |
| Glycine | 84.8 |
| Hypotaurine | 113.4 |
| Inositol, myo- | 115.9 |
| Lactic acid | 104.2 |
| Leucine | 106.6 |
| Malic acid | 126.9 |
| Pantothenic acid | 135.1 |
| Phenylalanine | 86.7 |
| Proline | 110.8 |
| Putrescine | 104.5 |
| Pyroglutamic acid | 119.5 |
| Pyruvic acid | 148.3 |
| Ribose | 125.6 |
| Serine | 119.6 |
| Succinic acid | 124.6 |
| Threonine | 128.2 |
| Uracil | 69.1 |
| Valine | 106.8 |

**Additional file 1**
Decoding the dynamics of cellular metabolism and the action of 3-bromopyruvate and 2-deoxyglucose using pulsed stable isotope-resolved metabolomics
Pietzke and Zasada et al., Cancer and Metabolism 2014

**Table S 6** – Intracellular metabolite concentrations of T98G cells.
Data is derived from the reproducibility experiment (see Methods section). T98G cells were incubated with $^{13}C_6$-glucose for three minutes.

| | **Quantity (pmol / $10^6$ cells)** | | | **$^{13}$C-Quantity (pmol / $10^6$ cells)** | | |
|---|---|---|---|---|---|---|
| **Compound** | **Average** | **±** | **STDEV** | **Average** | **±** | **STDEV** |
| Adenine | 77 | ± | 15 | *n.d* | ± | *n.d* |
| Alanine | 841 | ± | 89 | 10.3 | ± | 3.7 |
| Alanine, beta- | 623 | ± | 122 | *n.d* | ± | *n.d* |
| Asparagine | 202 | ± | 15 | *n.d* | ± | *n.d* |
| Aspartic acid | 251 | ± | 22 | 0.4 | ± | 0.5 |
| Butanoic acid, 2-amino | 3,965 | ± | 702 | *n.d* | ± | *n.d* |
| Butanoic acid, 3-hydroxy | 51 | ± | 7 | *n.d* | ± | *n.d* |
| Citric acid | 327 | ± | 17 | 3.7 | ± | 2 |
| Fructose | 1,068 | ± | 32 | 29.0 | ± | 2.6 |
| Fructose-1,6,diphosphate | 773 | ± | 45 | *n.d* | ± | *n.d* |
| Fructose-6-phosphate | 48 | ± | 5 | 10.8 | ± | 0.6 |
| Fumaric acid | 135 | ± | 6 | 0.4 | ± | 0.2 |
| Glucose-6-phosphate | 532 | ± | 27 | 100.6 | ± | 6.3 |
| Glutaric acid, 2-hydroxy | 98 | ± | 5 | 0.5 | ± | 0.3 |
| Glutaric acid, 2-oxo | 235 | ± | 6 | 2.3 | ± | 1.5 |
| Glyceric acid | 30 | ± | 2 | *n.d* | ± | *n.d* |
| Glyceric-acid-3-phosphate | 133 | ± | 7 | *n.d* | ± | *n.d* |
| Glycerol-3-phosphate | 90 | ± | 5 | 1.6 | ± | 0.7 |
| Glycine | 2,332 | ± | 135 | 4.0 | ± | 2.4 |
| Inositol, myo | 1,203 | ± | 20 | *n.d* | ± | *n.d* |
| Isoleucine | 1,960 | ± | 170 | *n.d* | ± | *n.d* |
| Lactic acid | 9,873 | ± | 1,269 | 454.5 | ± | 69.9 |
| Leucine | 2,409 | ± | 134 | *n.d* | ± | *n.d* |
| Lysine | 1,512 | ± | 238 | *n.d* | ± | *n.d* |
| Malic acid | 547 | ± | 22 | 1.2 | ± | 1.1 |
| Methionine | 1,009 | ± | 74 | *n.d* | ± | *n.d* |
| Pantothenic acid | 142 | ± | 3 | *n.d* | ± | *n.d* |
| Proline | 892 | ± | 56 | *n.d* | ± | *n.d* |
| Putrescine | 36 | ± | 1 | *n.d* | ± | *n.d* |
| Pyruvic acid | 195 | ± | 31 | 9.3 | ± | 1.5 |
| Ribose-5-phosphate | 185 | ± | 11 | *n.d* | ± | *n.d* |
| Serine | 2,546 | ± | 154 | *n.d* | ± | *n.d* |
| Succinic acid | 30 | ± | 8 | 0.1 | ± | 0.1 |
| Threonine | 4,971 | ± | 339 | *n.d* | ± | *n.d* |
| Tyrosine | 1,101 | ± | 173 | *n.d* | ± | *n.d* |
| Uracil | 111 | ± | 21 | *n.d* | ± | *n.d* |
| Uridine-5-monophosphate | 8,410 | ± | 615 | *n.d* | ± | *n.d* |
| Valine | 333 | ± | 17 | *n.d* | ± | *n.d* |

**Table S 7** – Metabolite specific mass fragments for the calculation of $^{13}C$-isotope incorporation. Mass fragments are chosen concerning their uniqueness for the derivate of the metabolite and were evaluated in separately performed $^{13}C_6$-glucose and $^{13}C_5$-glutamine labeling experiments.

| | | | Mass fragment (m/z) | | |
|---|---|---|---|---|---|
| **Compound** | **Derivate** | **Abbr.** | **Unlabeled** | **Labeling with u-$^{13}C$-glucose** | **Labeling with u-$^{13}C$-glutamine** |
| Alanine | 3TMS | Ala | 188 | 190 | - |
| Aspartic acid | 3TMS | Asp | 232 | 235 (a) | - |
| Citric acid | 4TMS | Cit | 273 | 275 | 277 |
| Dihydroxyacetone phosphate | 1MeOX 3TMS | DHAP | 400 | 403 | - |
| Fructose | 1MeOX 5TMS | Fru | 217 | 220 | - |
| Fructose-1,6-bisphosphate | 1MeOX 7TMS | F16BP | 217 | 220 | - |
| Fructose-6-phosphate | 1MeOX 6TMS | F6P | 217 | 220 | - |
| Fumaric acid | 2TMS | Fum | 245 | 247 | 249 |
| Glucose | 1MeOX 5TMS | Glc | 319 | 323 | - |
| Glucose-6-phosphate | 1MeOX 6TMS | G6P | 217 | 220 | - |
| Gluconic acid-6-phosphate | 7TMS | PG6 | 217 | 220 | - |
| Glutamic acid | 3TMS | Glu | 246 | - | 250 |
| Glutamine | 3TMS | Gln | 156 | - | 160 |
| Glutaric acid | 2TMS | Glut | 261 | - | 266 |
| Glutaric acid, 2-hydroxy | 3TMS | Glut-OH | 247 | - | 251 |
| Glutaric acid, 2-oxo | 1MeOX 2TMS | aKG | 198 | 200 | 203 |
| Glyceric acid-3-phosphate | 4TMS | 3PGA | 357 | 359 | - |
| Glycerol | 3TMS | Glyc | 218 | 221 | - |
| Glycerol-3-phosphate | 4TMS | Glyc3P | 357 | 359 | - |
| Glycine | 3TMS | Gly | 276 | 277 | - |
| Lactic acid | 2TMS | Lac | 219 | 222 | - |
| Malic acid | 3TMS | Mal | 233 | 235 | 236 |
| Phosphoenolpyruvic acid | 3TMS | PEP | 369 | 372 | - |
| Pyruvic acid | 1MeOX 1TMS | Pyr | 174 | 177 | - |
| Ribose-5-P | 1MeOX 5TMS | R5P | 217 | 220 | - |
| Serine | 3TMS | Ser | 204 | 206 | - |
| Succinic acid | 2TMS | Suc | 247 | 249 | 251 |

(a) – when simultaneously applied with $^{13}C$-glutamine labeling, the resulting $^{13}C$-pyroglutamate might interfere with this mass range.

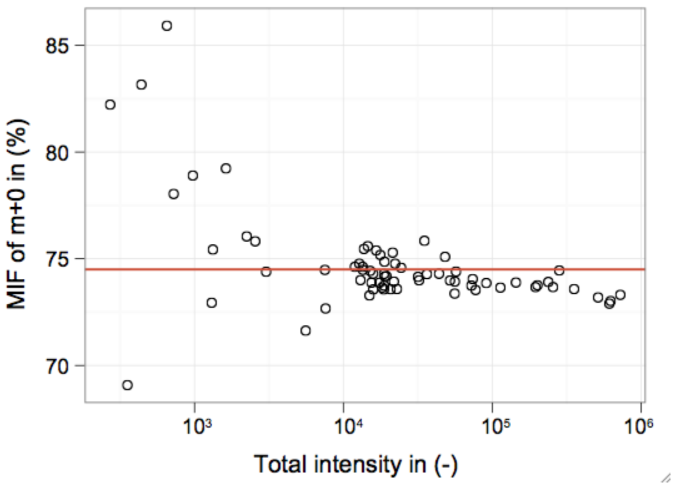


**Figure S 4 -** Concentration dependency of mass isotopomer fractions.
The mass isotopomer fraction (MIF) of mass fragment m+0 for citric acid (m/z = 273-279) is shown for a broad intensity range (black circles). The expected value (red line) was calculated by Mass Spec Calculator Pro Demo version 4.09 (ChemSW, Inc.). The proportion of m+0 in the mass range decreases and yields higher precision with increasing levels of citric acid.

**Additional file 1**
Decoding the dynamics of cellular metabolism and the action of 3-bromopyruvate and 2-deoxyglucose using pulsed stable isotope-resolved metabolomics
Pietzke and Zasada et al., Cancer and Metabolism 2014

**Table S 8** – Illustration of the position dependent strategy for correction of natural $^{13}C$-isotope abundance.

$^{13}C_1$-Glucose and $^{12}C$-glucose were mixed in known ratios to proof the position dependent (targeted) calculation strategy. Two examples are shown for the determination of $^{13}C$-incorporation as described.

| | | Intensities in (-) | | | MIF in (%) | | | 10 % $^{13}C_1$ | | | 50 % $^{13}C_1$ | | |
|---|---|---|---|---|---|---|---|---|---|---|---|---|---|
| **m/z** | | **$^{12}C$** | **10 % $^{13}C_1$** | **50 % $^{13}C_1$** | **$^{12}C$** | **10 % $^{13}C_1$** | **50 % $^{13}C_1$** | **$S_{nat+1}$** | **$S_{inc+1}$** | **L** | **$S_{nat+1}$** | **$S_{inc+1}$** | **L** |
| 319 | m0 | 156460 | 153425 | 84507 | 66.6 | 60.2 | 33.2 | | | | | | |
| **320** | **m+1** | **48250** | **63669** | **112421** | **20.6** | **25.0** | **44.2** | **18.6** | **6.4** | **9.6** | **10.2** | **33.9** | **50.5** |
| 321 | m+2 | 23579 | 28289 | 38071 | 10.0 | 11.1 | 15.0 | 9.1 | 2.0 | | 5.0 | 10.0 | |
| 322 | m+3 | 5038 | 7389 | 15467 | 2.1 | 2.9 | 6.1 | 1.9 | 1.0 | | 1.1 | 5.0 | |
| 323 | m+4 | 1199 | 1716 | 3268 | 0.5 | 0.7 | 1.3 | 0.5 | 0.2 | | 0.3 | 1.0 | |
| 324 | m+5 | 228 | 262 | 751 | 0.1 | 0.1 | 0.3 | 0.1 | 0.0 | | 0.0 | 0.2 | |
| | Sum | 234754 | 254750 | 254485 | 100 | 100 | 100 | | | | | | |

**Additional file 1**
Decoding the dynamics of cellular metabolism and the action of 3-bromopyruvate and 2-deoxyglucose using pulsed stable isotope-resolved metabolomics
Pietzke and Zasada et al., Cancer and Metabolism 2014

**Table S 9** – Validation of correction strategies for the natural $^{13}C$-carbon abundance. 1-$^{13}C_1$-Glucose (purity 99%) and unlabeled glucose were mixed in known ratios and measured in three independent replicates. Uncorrected values overestimate incorporation especially in the lower range of isotope incorporation, whereas both calculation strategies consider the natural contribution of carbon-13 correctly.

| Expected isotope abundance (%) | $^{13}C$-abundance in glucose (%) | | |
|---|---|---|---|
| | **Uncorrected** | **Targeted** | **Position-independent** |
| 0 | 23.4 ± 0.09 | 0.0 ± 0.16 | 0.0 ± 0.12 |
| 1.98 | 24.7 ± 0.25 | 2.1 ± 0.42 | 2.0 ± 0.32 |
| 4.95 | 26.4 ± 0.21 | 5.0 ± 0.36 | 5.0 ± 0.27 |
| 9.90 | 29.6 ± 0.20 | 10.3 ± 0.33 | 10.1 ± 0.29 |
| 24.75 | 39.2 ± 0.79 | 25.3 ± 1.19 | 25.0 ± 1.26 |
| 49.50 | 56.8 ± 0.89 | 50.3 ± 1.18 | 49.8 ± 1.28 |
| 74.25 | 76.1 ± 0.78 | 74.2 ± 0.91 | 74.0 ± 0.94 |
| 89.10 | 89.0 ± 0.04 | 88.6 ± 0.04 | 88.4 ± 0.03 |
| 94.05 | 93.9 ± 0.14 | 93.8 ± 0.15 | 93.7 ± 0.15 |
| 97.02 | 96.8 ± 0.19 | 96.7 ± 0.19 | 96.7 ± 0.20 |
| 99.00 | 98.7 ± 0.04 | 98.7 ± 0.04 | 98.7 ± 0.05 |

**Table S 10** – Biological and technical variation of $^{13}$C-glucose incorporation.
T98G cells were incubated with u-$^{13}$C glucose for three minutes and harvested as described in the Methods section.

| | | Average ($^{13}$C-Glc incorporation in %) ± STDEV | | | | | |
|---|---|---|---|---|---|---|---|
| **Compound** | **Mass** | **Technical repl.** | | | **Biological repl.** | | |
| 3PGA | 357 | 77.7 | ± | 4.0 | 83.2 | ± | 2.1 |
| Ala | 188 | 4.7 | ± | 1.3 | 5.5 | ± | 0.6 |
| Cit | 273 | 4.2 | ± | 2.0 | 7.1 | ± | 1.2 |
| F16BP | 217 | 78.8 | ± | 1.0 | 79.7 | ± | 0.7 |
| Fru | 217 | 10.7 | ± | 0.7 | 7.7 | ± | 0.7 |
| F6P | 217 | 84.0 | ± | 4.8 | 83.2 | ± | 4.0 |
| Fum | 245 | 0.9 | ± | 0.7 | 0.6 | ± | 0.2 |
| G6P | 217 | 74.6 | ± | 0.5 | 74.3 | ± | 0.4 |
| Glut-OH | 198 | 3.2 | ± | 2.4 | 1.5 | ± | 1.6 |
| Glyc3P | 357 | 6.7 | ± | 2.7 | 8.9 | ± | 1.3 |
| Gly | 276 | 0.2 | ± | 0.6 | *n.d.* | ± | *n.d.* |
| Lac | 219 | 17.9 | ± | 0.4 | 18.4 | ± | 2.7 |
| Mal | 245 | 0.7 | ± | 0.7 | 0.2 | ± | 1.1 |
| Pyr | 174 | 18.9 | ± | 3.0 | 17.3 | ± | 1.3 |
| Suc | 247 | *n.d.* | ± | *n.d.* | 1.0 | ± | 1.2 |

**Additional file 1**
Decoding the dynamics of cellular metabolism and the action of 3-bromopyruvate and 2-deoxyglucose using pulsed stable isotope-resolved metabolomics
Pietzke and Zasada et al., Cancer and Metabolism 2014

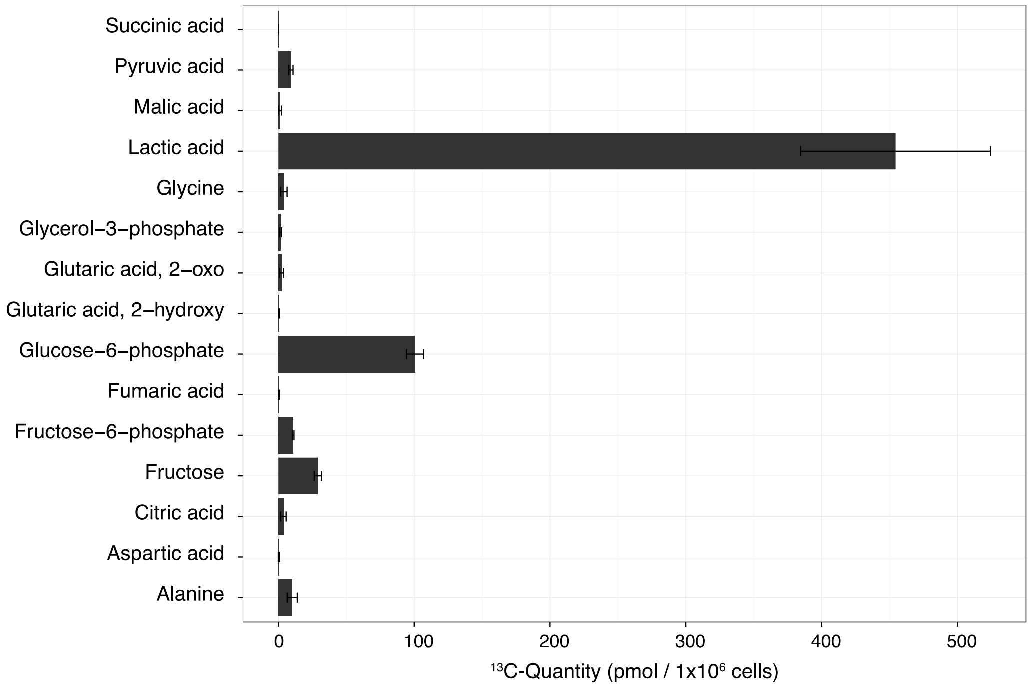


**Figure S 5 -** Absolute $^{13}$C-quantities of intracellular metabolites in T98G cells.
Data derived from the reproducibility experiment (see Methods section and supplement table 6).

**Additional file 1**
Decoding the dynamics of cellular metabolism and the action of 3-bromopyruvate and 2-deoxyglucose using pulsed stable isotope-resolved metabolomics
Pietzke and Zasada et al., Cancer and Metabolism 2014

**Table S 11** – Comparison of carbon routing within the CCM of different cell lines.
The labeled quantities of metabolites after $^{13}$C-Glucose incubation into T98G, HEK293, HeLa and HCT-116 cells reveal differences in carbon routing in the central carbon metabolism. Averages and standard deviations for labeled quantities are shown relative to T98G cells.

| | **$^{13}$C-Glucose labeled quantity Average ± STDEV** | | | | | | | | | | | |
|---|---|---|---|---|---|---|---|---|---|---|---|---|
| **Compound** | **T98G** | | | **HEK293** | | | **HeLa** | | | **HCT-116** | | |
| 3PGA | 1.00 | ± | 0.04 | 2.77 | ± | 0.08 | 1.98 | ± | 0.01 | 2.47 | ± | 0.01 |
| Ala | 1.00 | ± | 0.22 | 9.59 | ± | 2.65 | 2.53 | ± | 0.65 | 1.62 | ± | 0.63 |
| Cit | 1.00 | ± | 0.20 | 13.32 | ± | 0.32 | 9.82 | ± | 0.34 | 17.66 | ± | 0.35 |
| DHAP | 1.00 | ± | 0.08 | 2.51 | ± | 0.26 | 1.68 | ± | 0.30 | 3.04 | ± | 0.10 |
| F1P | 1.00 | ± | 0.15 | 0.84 | ± | 0.06 | 0.95 | ± | 0.21 | 65.64 | ± | 0.07 |
| F6P | 1.00 | ± | 0.06 | 1.11 | ± | 0.02 | 0.73 | ± | 0.05 | 10.89 | ± | 0.05 |
| Fru | 1.00 | ± | 0.04 | 0.32 | ± | 0.01 | 9.56 | ± | 0.04 | 12.40 | ± | 0.58 |
| G6P | 1.00 | ± | 0.07 | 3.75 | ± | 0.02 | 2.34 | ± | 0.04 | 6.82 | ± | 0.06 |
| Glyc | 1.00 | ± | 0.03 | 0.67 | ± | 0.12 | 3.96 | ± | 0.09 | 4.40 | ± | 0.58 |
| Glyc3P | 1.00 | ± | 0.09 | 15.57 | ± | 0.86 | 1.85 | ± | 0.40 | 3.78 | ± | 0.00 |
| Lac | 1.00 | ± | 0.04 | 8.88 | ± | 2.56 | 6.64 | ± | 0.02 | 12.78 | ± | 0.53 |
| Pyr | 1.00 | ± | 0.02 | 8.34 | ± | 1.10 | 4.18 | ± | 0.80 | 10.65 | ± | 0.24 |
| R5P | 1.00 | ± | 0.12 | 1.21 | ± | 0.13 | 1.21 | ± | 0.07 | 1.52 | ± | 0.01 |
| Ser | 1.00 | ± | 0.21 | 17.29 | ± | 1.68 | 5.03 | ± | 0.61 | 7.06 | ± | 0.93 |

**Additional file 1**

Decoding the dynamics of cellular metabolism and the action of 3-bromopyruvate and 2-deoxyglucose using pulsed stable isotope-resolved metabolomics

Pietzke and Zasada et al., Cancer and Metabolism 2014

**Table S 12** – 3-Bromopyruvate treatment rearranges central carbon metabolism in T98G cells.
T98G cells were treated for 12 min with 2 mM 3-Bromopyruvate and labeled for 3 minutes with u-$^{13}$C-glucose. Fold changes of peak intensity and $^{13}$C-label incorporation are shown relative to control. Two-sided, heteroscedastic t-test indicates significant changes (+++ < 0.005, ++ < 0.01, + < 0.05, n.s. = not significant, *n.d.* = not detected).

| | **Fold changes relative to control** | | | | | | **% Change relative to control** | | | | | | **T-Test** | | | | | |
|---|---|---|---|---|---|---|---|---|---|---|---|---|---|---|---|---|---|---|
| **Compound** | **Peak intensity** | | **Label incorporation** | | **Label inc. x intensity** | | **Peak intensity** | | **Label incorporation** | | **Label inc. x intensity** | | **Peak intensity** | | **Label incorporation** | | **Label inc. x intensity** | |
| | **15min** | **30 min** | **15min** | **30 min** | **15min** | **30 min** | **15min** | **30 min** | **15min** | **30 min** | **15min** | **30 min** | **15min** | **30 min** | **15min** | **30 min** | **15min** | **30 min** |
| G6P | 0,8 | 1,7 | 0,34 | 0,25 | 0,28 | 0,43 | -17 | 68 | -66 | -75 | -72 | -57 | n.s. | +++ | +++ | +++ | +++ | +++ |
| F6P | 3,2 | 8,2 | 0,10 | 0,07 | 0,31 | 0,55 | 224 | 722 | -90 | -93 | -69 | -45 | +++ | +++ | +++ | +++ | +++ | +++ |
| F1,6-BP | 3,9 | 3,4 | 0,37 | 0,44 | 1,07 | 1,68 | 285 | 240 | -63 | -56 | 6,8 | 68,0 | ++ | + | ++ | +++ | n.s. | + |
| F1P | 0,7 | 1,2 | 1,13 | 0,70 | 0,57 | 0,59 | -26 | 21 | 13 | -30 | -43 | -41 | + | n.s. | n.s. | + | n.s. | n.s. |
| Fru | 1,0 | 1,1 | 0,30 | 0,30 | 0,30 | 0,34 | 1 | 11 | -70 | -70 | -70 | -66 | n.s. | n.s. | +++ | +++ | +++ | +++ |
| 6PG | 4,5 | 5,1 | *n.d.* | *n.d.* | *n.d.* | *n.d.* | 355 | 413 | *n.d.* | *n.d.* | *n.d.* | *n.d.* | +++ | +++ | | | | |
| 3PGA | *n.d.* | 0,3 | *n.d.* | 0,07 | *n.d.* | 0,03 | *n.d.* | -69 | *n.d.* | -93 | *n.d.* | -96,5 | | +++ | | | | |
| Pyr | 1,0 | 1,0 | 0,10 | 0,05 | 0,05 | 0,04 | -2 | -1 | -90 | -95 | -94,8 | -95,8 | n.s. | n.s. | +++ | +++ | +++ | +++ |
| Lac | 1,1 | 0,9 | 0,036 | 0,028 | 0,039 | 0,025 | 6 | -9 | -96,4 | -97,2 | -96,1 | -97,5 | n.s. | n.s. | +++ | +++ | +++ | +++ |
| Glyc3P | 0,8 | 0,7 | *n.d.* | *n.d.* | *n.d.* | n.d | -16 | -28 | *n.d.* | *n.d.* | *n.d.* | *n.d.* | n.s. | +++ | | | | |
| Glyc | 1,1 | 1,1 | *n.d.* | *n.d.* | *n.d.* | n.d | 12 | 7 | *n.d.* | n.d | *n.d.* | n.d | +++ | + | | | | |
| Ala | 3,6 | 4,2 | 0,05 | 0,07 | 0,19 | 0,23 | 257 | 324 | -95 | -93 | -81 | -77 | +++ | +++ | +++ | +++ | +++ | +++ |
| Ser | 0,8 | 1,1 | *n.d.* | *n.d.* | *n.d.* | *n.d.* | -20 | 8 | *n.d.* | *n.d.* | *n.d.* | *n.d.* | n.s. | n.s. | | | | |
| Cit | 0,5 | 0,8 | 0,094 | 0,11 | 0,06 | 0,12 | -49 | -24 | -90,6 | -89 | -94,1 | -87,6 | +++ | n.s. | +++ | +++ | +++ | +++ |
| aKG | 1,4 | 1,6 | *n.d.* | *n.d.* | *n.d.* | n.d | 40 | 58 | *n.d.* | n.d | *n.d.* | n.d | +++ | +++ | | | | |
| Succ | 1,2 | 1,0 | *n.d.* | *n.d.* | *n.d.* | n.d | 17 | 4 | *n.d.* | n.d | *n.d.* | n.d | ++ | n.s. | | | | |
| Fum | 0,4 | 0,4 | *n.d.* | *n.d.* | *n.d.* | n.d | -64 | -64 | *n.d.* | n.d | *n.d.* | n.d | +++ | +++ | | | | |
| Mal | 0,3 | 0,3 | *n.d.* | *n.d.* | *n.d.* | n.d | -72 | -69 | *n.d.* | n.d | *n.d.* | n.d | +++ | +++ | | | | |

**Additional file 1**

Decoding the dynamics of cellular metabolism and the action of 3-bromopyruvate and 2-deoxyglucose using pulsed stable isotope-resolved metabolomics

Pietzke and Zasada et al., Cancer and Metabolism 2014

**Table S 13** – Effect of 2-Deoxyglucose on the central carbon metabolism of T98G cells.

T98G cells were incubated with two different concentrations of 2-Deoxyglucose for 15 min. Rearrangement of metabolism were monitored by $^{13}$C-glucose incorporation for 3 minutes. Fold changes of peak intensity and $^{13}$C-label incorporation are shown relative to control. Two-sided, heteroscedastic t-test indicates significant changes (+++ < 0.005, ++ < 0.01, + < 0.05, n.s. = not significant, *n.d.* = not detected).

| | **Fold changes relative to control** | | | | | | **% Change relative to control** | | | | | | **T-Test** | | | | | |
|---|---|---|---|---|---|---|---|---|---|---|---|---|---|---|---|---|---|---|
| **Compound** | **Peak intensity** | | **Label Inc.** | | **Label Inc. x intensity** | | **Peak intensity** | | **Label Inc.** | | **Label Inc. x intensity** | | **Peak intensity** | | **Label Inc.** | | **Label Inc. x intensity** | |
| | **2mM** | **10mM** | **2mM** | **10mM** | **2mM** | **10mM** | **2mM** | **10mM** | **2mM** | **10mM** | **2mM** | **10mM** | **2mM** | **10mM** | **2mM** | **10mM** | **2mM** | **10mM** |
| G6P | 0.8 | 0.5 | 1.0 | 0.9 | 0.7 | 0.5 | -24 | -49 | -4.9 | -6.3 | -28 | -53 | n.s. | ++ | n.s. | n.s. | + | +++ |
| F6P | 0.7 | 0.5 | 1.1 | 1.0 | 0.8 | 0.6 | -27 | -46 | 7.8 | 5.0 | -20 | -40 | n.s. | n.s. | n.s. | n.s. | n.s. | n.s. |
| Fru | 1.1 | 1.0 | 0.9 | 0.8 | 1.0 | 0.8 | 7.9 | -0.2 | -10.6 | -21.7 | -3.3 | -22.5 | n.s. | n.s. | n.s. | n.s. | n.s. | ++ |
| 3PGA | 0.8 | *n.d.* | 1.1 | *n.d.* | 0.9 | *n.d.* | -16.9 | *n.d.* | 6.6 | *n.d.* | -11.2 | *n.d.* | n.s. | | n.s. | | n.s. | |
| Pyr | 0.7 | 0.3 | 1.3 | 0.8 | 0.9 | 0.2 | -28.7 | -71.1 | 31.3 | -21.8 | -6.8 | -79.0 | + | +++ | +++ | n.s. | n.s. | +++ |
| Lac | 0.8 | 0.7 | 1.2 | 0.8 | 1.0 | 0.6 | -19.6 | -28.5 | 20.2 | -17.5 | -4.7 | -41.0 | n.s. | +++ | n.s. | + | n.s. | +++ |
| Glyc3P | 0.9 | 1.1 | 1.8 | 1.3 | 1.7 | 1.6 | -8.1 | 14.3 | 78.2 | 26.6 | 72.9 | 57.2 | n.s. | n.s. | + | n.s. | n.s. | n.s. |
| Glyc | 1.0 | 1.1 | *n.d.* | *n.d.* | *n.d.* | *n.d.* | -3.8 | 5.3 | *n.d.* | *n.d.* | *n.d.* | *n.d.* | n.s. | n.s. | | | | |
| Ala | 1.1 | 0.8 | 1.7 | 1.1 | 1.3 | 0.6 | 11.0 | -15.8 | 70.2 | 5.3 | 31.9 | -44.6 | n.s. | n.s. | n.s. | n.s. | n.s. | n.s. |
| Ser | 0.8 | 0.7 | *n.d.* | *n.d.* | *n.d.* | *n.d.* | -17.7 | -31.8 | *n.d.* | *n.d.* | *n.d.* | *n.d.* | n.s. | + | n.s. | n.s. | | |
| Cit | 1.3 | 1.9 | 1.7 | 1.4 | 2.2 | 2.5 | 30.0 | 85.6 | 66.7 | 36.8 | 115.4 | 152.6 | n.s. | +++ | +++ | n.s. | +++ | +++ |
| aKG | 1.0 | 0.5 | *n.d.* | *n.d.* | *n.d.* | n.d | -0.1 | -47.5 | *n.d.* | n.d | *n.d.* | n.d | n.s. | ++ | | | | |
| Succ | 0.8 | 0.8 | *n.d.* | *n.d.* | *n.d.* | n.d | -17.3 | -15.3 | *n.d.* | n.d | *n.d.* | n.d | + | + | | | | |
| Fum | 0.9 | 0.6 | 1.1 | 1.0 | 1.0 | 0.6 | -6.2 | -38.9 | 13.1 | -2.9 | 3.1 | -41.5 | n.s. | +++ | n.s. | n.s. | n.s. | n.s. |
| Mal | 0.9 | 0.6 | 1.4 | 1.1 | 1.2 | 0.6 | -7.2 | -40.6 | 37.2 | 11.3 | 23.1 | -35.8 | n.s. | +++ | n.s. | n.s. | n.s. | n.s. |

**Additional file 1**
Decoding the dynamics of cellular metabolism and the action of 3-bromopyruvate and 2-deoxyglucose using pulsed stable isotope-resolved metabolomics
Pietzke and Zasada et al., Cancer and Metabolism 2014

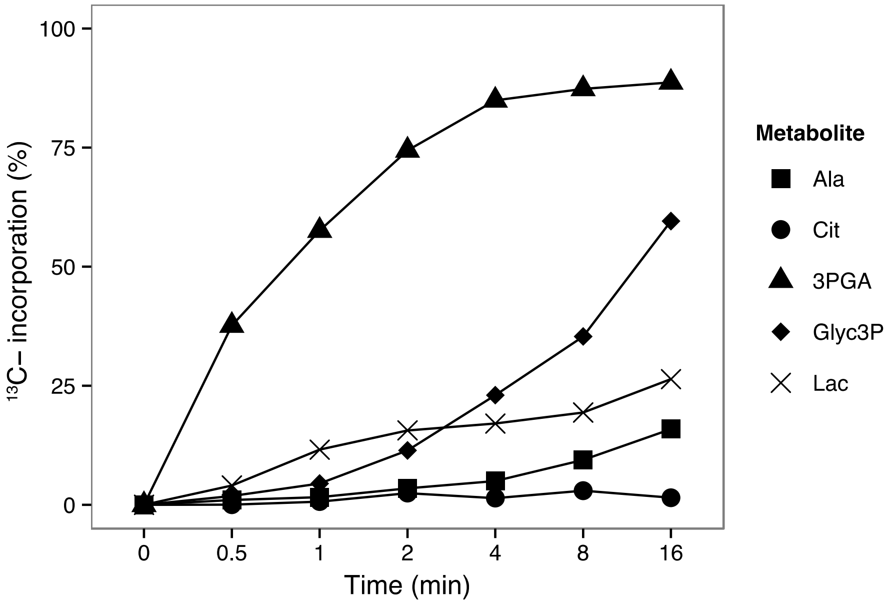


**Figure S 6 -** Time dependency of $^{13}$C-label incorporation into intermediates of the central carbon metabolism.
HEK293 cells, grown in DMEM supplemented with 10 % FBS, 2.5 g/L glucose, and 4 mM glutamine, were incubated with $^{13}$C-glucose up to 16 min. Harvest, metabolite extraction, GC-MS measurement and determination of $^{13}$C-label incorporation were done as described in the Methods section.

**Additional file 1**
Decoding the dynamics of cellular metabolism and the action of 3-bromopyruvate and 2-deoxyglucose using pulsed stable isotope-resolved metabolomics
Pietzke and Zasada et al., Cancer and Metabolism 2014

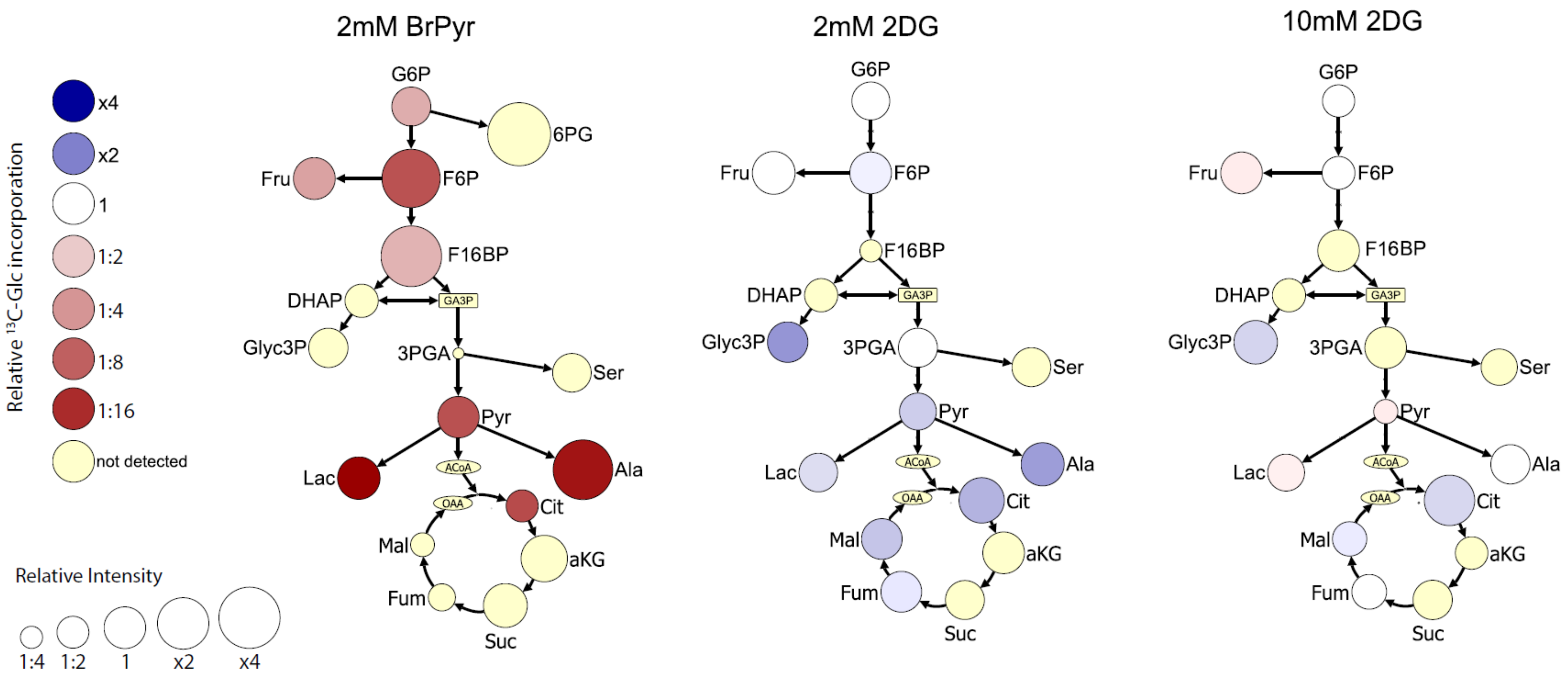


**Figure S 7** – Graphical representation of small molecule inhibitor induced rearrangement of central carbon metabolism.
The incorporation of $^{13}$C-glucose and abundance of metabolites are presented by color or size relative to the untreated control after 15 minutes of inhibitor treatment and 3 min of $^{13}$C-glucose incubation.

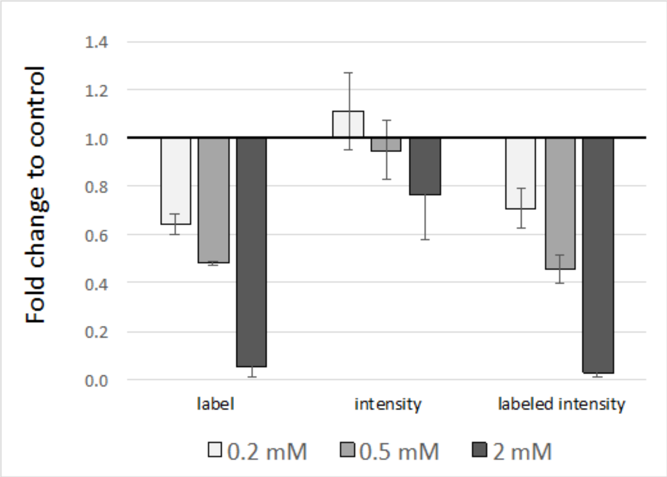


**Figure S 8 -** Concentration dependency of BrPyr induced inhibition in carbon flow from $^{13}C$-glucose to lactate.

Three different concentrations of 3-Bromopyruvate were applied for 15 minutes to T98G cells and incorporation of u-$^{13}C$-glucose was monitored for 3 minutes in 3 biological replicates. Fold change of incorporated label, intensity (peak area) and labeled intensity were plotted relative to an untreated control.

**Additional file 1**
Decoding the dynamics of cellular metabolism and the action of 3-bromopyruvate and 2-deoxyglucose using pulsed stable isotope-resolved metabolomics
Pietzke and Zasada et al., Cancer and Metabolism 2014

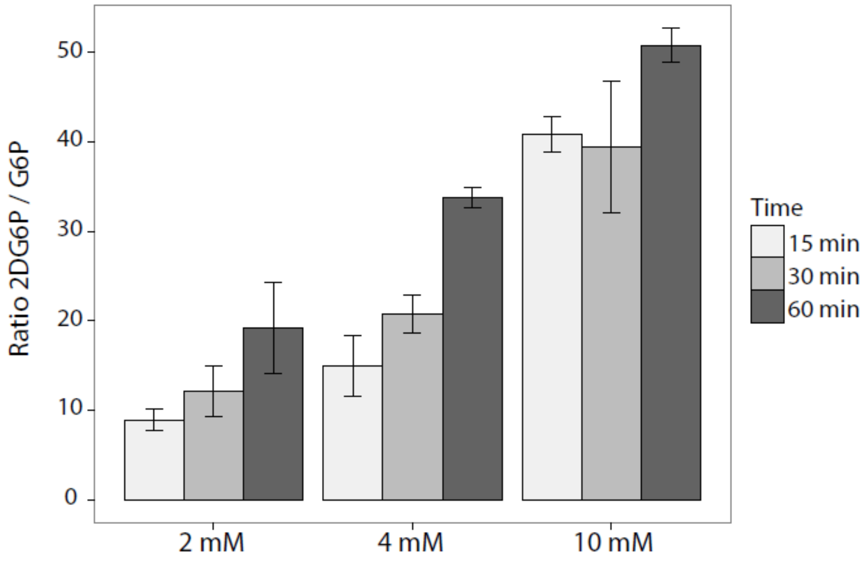


**Figure S** 9 - Time and concentration dependent accumulation of 2-deoxyglucose-6-phosphate (2DG-P). Shown are averages of ratios of two biological replicates measured in two technical replicates.
